# Multiple mechanisms explain loss of anthocyanins from betalain‐pigmented Caryophyllales, including repeated wholesale loss of a key anthocyanidin synthesis enzyme

**DOI:** 10.1111/nph.19341

**Published:** 2023-10-28

**Authors:** Boas Pucker, Nathanael Walker‐Hale, Jasmina Dzurlic, Won C. Yim, John C. Cushman, Alexandra Crum, Ya Yang, Samuel F. Brockington

**Affiliations:** ^1^ Department of Plant Sciences University of Cambridge Cambridge CB2 3EA UK; ^2^ Plant Biotechnology and Bioinformatics, Institute of Plant Biology & BRICS TU Braunschweig 38106 Braunschweig Germany; ^3^ Department of Biochemistry & Molecular Biology University of Nevada Reno NV 89557 USA; ^4^ Department of Plant and Microbial Biology University of Minnesota‐Twin Cities St Paul MN 55108 USA

**Keywords:** AN9, anthocyanin biosynthesis, betalain biosynthesis, flavonoid biosynthesis, MBW complex, PAP1, pigment evolution, TT19

## Abstract

- In this study, we investigate the genetic mechanisms responsible for the loss of anthocyanins in betalain‐pigmented Caryophyllales, considering our hypothesis of multiple transitions to betalain pigmentation.
- Utilizing transcriptomic and genomic datasets across 357 species and 31 families, we scrutinize 18 flavonoid pathway genes and six regulatory genes spanning four transitions to betalain pigmentation. We examined evidence for hypotheses of wholesale gene loss, modified gene function, altered gene expression, and degeneration of the MBW (MYB‐bHLH‐WD40) trasnscription factor complex, within betalain‐pigmented lineages.
- Our analyses reveal that most flavonoid synthesis genes remain conserved in betalain‐pigmented lineages, with the notable exception of *TT19* orthologs, essential for the final step in anthocyanidin synthesis, which appear to have been repeatedly and entirely lost. Additional late‐stage flavonoid pathway genes upstream of *TT19* also manifest strikingly reduced expression in betalain‐pigmented species. Additionally, we find repeated loss and alteration in the MBW transcription complex essential for canonical anthocyanin synthesis.
- Consequently, the loss and exclusion of anthocyanins in betalain‐pigmented species appear to be orchestrated through several mechanisms: loss of a key enzyme, downregulation of synthesis genes, and degeneration of regulatory complexes. These changes have occurred iteratively in Caryophyllales, often coinciding with evolutionary transitions to betalain pigmentation.

## Introduction

The tree of life is replete with polyphyletic traits, a phenomenon also known as homoplasy, which may be explained by iterative origins of a trait, trait loss, or reversals to an ancestral state (Wake *et al*., 2011). Resolving these alternative scenarios is challenging, but a promising approach lies in a detailed genetic examination of the trait in question. For example, the homoplastic pattern of differentiated perianth in Ranunculales, initially ascribed to convergent evolution of differentiation, has now been shown to be derived through repeated petal loss via iterative loss or downregulation of the petal‐specific developmental regulator AP3‐3 (Rasmussen *et al*., 2009; Sharma *et al*., 2011; Zhang *et al*., 2013). Likewise, phylogenomic analysis of the NIN transcription factor, crucial for root nodule symbiosis, revealed multiple loss‐of‐function events that emphasize homoplasy via multiple losses (Griesmann *et al*., 2018), rather than iterated gains in symbiosis as previously hypothesized (Doyle, 1998). In a final example, initial suggestions of repeated origins of stomata (Pressel *et al*., 2014) have been ruled out following examination of patterns of gene conservation and loss, which posit a single origin of stomata and subsequent loss in liverworts (Chater *et al*., 2017; Harris *et al*., 2020). Phylogenetic analysis of underlying genetic variation therefore serves both as a robust tool to elucidate the evolutionary history of traits and to identify potential genetic mechanisms underlying trait transitions.

With this as context, anthocyanin pigmentation has been developed into a powerful model in which to explore the interaction between genetic architecture and trait transitions. Anthocyanin‐based floral coloration shows frequent transitions between different hues through changes in concentrations of red pelargonidins, purple cyanidins, and blue delphinidins (Wessinger & Rausher, 2012; Wheeler *et al*., 2021). These transitions can be caused by repeated regulatory changes to the same or similar anthocyanin pathway genes (Larter *et al*., 2018; Wheeler *et al*., 2023). The targets of these changes appear to be constrained by pleiotropy and the structure of the anthocyanin biosynthesis pathway (Streisfeld & Rausher, 2011; Larter *et al*., 2018; Wheeler *et al*., 2021). Some lineages have also repeatedly evolved white flowers by loss of anthocyanins (Ho & Smith, 2016; Wheeler *et al*., 2023), by diverse mechanisms (Gates *et al*., 2018; Duncan & Rausher, 2020), but with predictable molecular evolutionary consequences for genes in the pathway (Ho & Smith, 2016). Interestingly, while some transitions to white flowers involve gene loss or loss of function in anthocyanin pathway genes (Coburn *et al*., 2015; Lin *et al*., 2022), others seem to involve primarily regulatory changes (Ho & Smith, 2016; Gates *et al*., 2018), suggesting that some losses of anthocyanins may be more easily reversible. But while loss of anthocyanins in specific tissues is not uncommon, complete loss of anthocyanins is rare, and has only been documented in the angiosperm order Caryophyllales.

**Fig. 1:**
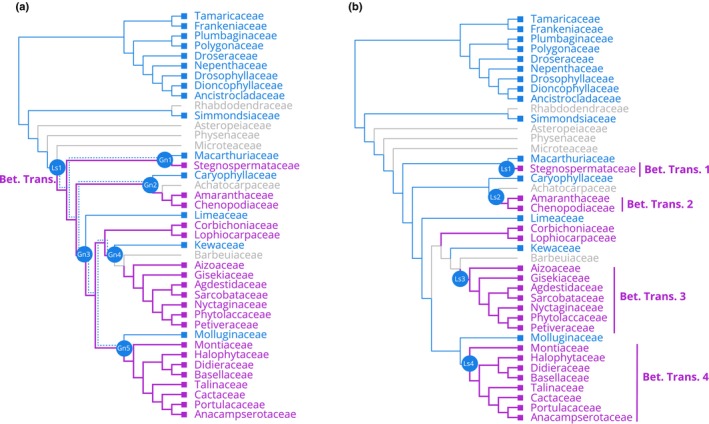
Two alternative hypotheses of pigment evolution in Caryophyllales. (a) A single origin of betalain pigmentation (*sensu* Brockington *et al*., 2015) implies a single loss of anthocyanins and subsequently five independent reversals (Gn1–5) back to anthocyanin pigmentation (dotted blue lines represent the maintenance of anthocyanin pathway genes); (b) in this scenario, all instances of anthocyanin pigmentation represent retention of the plesiomorphic state, and multiple transitions (Bet. Trans 1–4) to betalain pigmentation (se*nsu* Sheehan *et al*., 2020) implying at least four independent losses of anthocyanin (Ls1–4). Blue = anthocyanin, purple = betalain, gray = unknown. Tree topology and color coding based on the mutual exclusion between the betalain and anthocyanin pigmentation and the family level phylogeny of Sheehan *et al*. (2020).

In Caryophyllales, an unusual class of pigments, the betalains, replaces the otherwise ubiquitous anthocyanins. Intriguingly, species within Caryophyllales that exhibit betalain pigmentation have consistently shown an absence of anthocyanin pigments, and *vice versa* (Bate‐Smith, 1962; Mabry, 1964; Clement & Mabry, 1996). Despite this, betalain‐pigmented species continue to produce other flavonoids, such as proanthocyanidins in their seed coats (Shimada *et al*., 2005). These findings have led to the prevailing hypothesis that anthocyanins and betalains are mutually exclusive (Stafford, 1994; Clement & Mabry, 1996). Phylogenetic analyses reveal a complex, homoplastic distribution of betalain and anthocyanin pigmentation within Caryophyllales (Sheehan *et al*., 2020; Fig. 1a). This interdigitated pattern has traditionally been ascribed to a single origin of betalains early in the evolutionary history of Caryophyllales, followed by multiple reversals to anthocyanin pigmentation (Fig. 1a; Brockington *et al*., 2011, 2015). However, recent evidence challenges this view, suggesting that betalain synthesis has originated independently multiple times within Caryophyllales (Sheehan *et al*., 2020), which implies multiple independent losses of anthocyanins (Fig. 1b). Four distinct evolutionary genetic mechanisms can be hypothesized to explain the loss of anthocyanins in betalain‐pigmented Caryophyllales lineages: *Hypothesis 1* – wholesale loss of anthocyanin synthesis genes, *Hypothesis 2* – loss of function in anthocyanin synthesis genes, *Hypothesis 3* – reduced expression of anthocyanin synthesis genes, and *Hypothesis 4* – degeneration of *trans*‐acting factors responsible for the activation of anthocyanin synthesis genes.

**Fig. 2:**
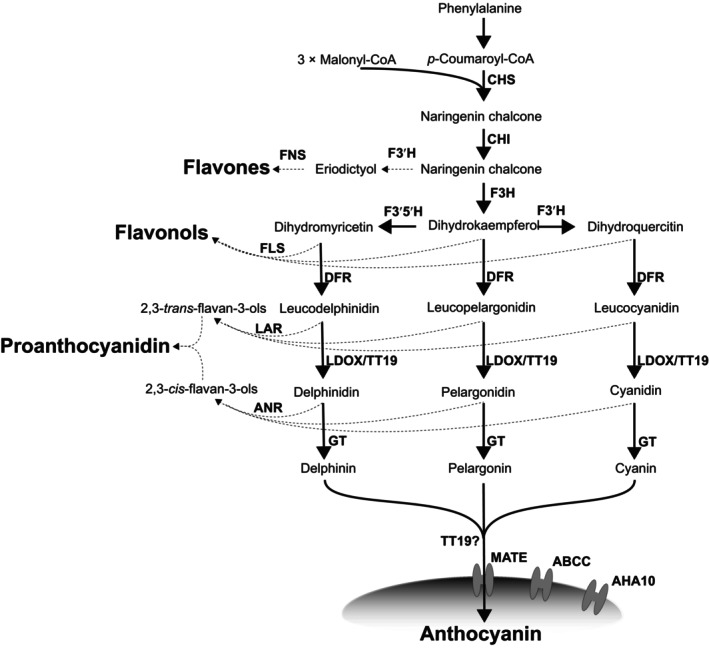
Simplified flavonoid biosynthesis pathway. CHS (naringenin‐chalcone synthase), CHI (chalcone isomerase), FNS (flavone synthase), FLS (flavonol synthase), F3H (flavanone 3‐hydroxylase), F3′H (flavonoid 3′‐hydroxylase), F3′5′H (flavonoid 3′,5′‐hydroxylase), DFR (dihydroflavonol 4‐reductase), LDOX (leucoanthocyanidin dioxygenase), TT19 (glutathione *S*‐transferase), LAR (leucoanthocyanidin reductase), ANR (anthocyanidin reductase), GT (glycosyltransferase; here the arrow represents glycosyltransferase enzymes in general rather than a specific glycosyltransferase, as glycosylations take place as series of steps), MATE (multidrug and toxin extrusion), ABCC (ATP binding cassette protein type C), and AHA10 (autoinhibited H(+)‐ATPase isoform 10). Note TT19 appears twice and with a ‘?’ in the second instance to reflect uncertainty in its role as a ligandin, in addition to its recently identified role in anthocyanidin synthesis (Eichenberger *et al*., 2023). MATE, ABCC, and AHA10 are involved in the anthocyanin transport from the cytoplasm into the vacuole. Shaded oval represents the vacuole in which anthocyanins are stored. Dotted lines show branch points from anthocyanin metabolism to other phenylalanine‐derived metabolites.

Studies examining the anthocyanin synthesis pathway (Fig. 2) in five betalain‐pigmented species have yielded some insights with respect to these hypotheses. First, current evidence does not support the notion of a wholesale loss of genes involved in anthocyanin synthesis. Specifically, the genes encoding late‐stage dihydroflavonol 4‐reductase (DFR) and leucoanthocyanidin dioxygenase (LDOX) are retained in betalain‐pigmented *Spinacia oleracea*, *Phytolacca americana*, and *Astrophytum myriostigma*. This conservation is likely attributable to the pleiotropic roles of DFR and ANS, which include proanthocyanidin synthesis (Shimada *et al*., 2004, 2005; Sakuta *et al*., 2021). Implicit then in the retention of these late‐stage genes is the preservation of preceding elements within the pathway. Second, evidence regarding the loss of function in anthocyanin synthesis genes remains inconclusive. While canonical functions for ANS and DFR are conserved in *S. oleracea* and *P. americana* (Shimada *et al*., 2004, 2005; Sakuta *et al*., 2021), Polturak *et al*. (2018) identified a truncated ANS protein in *Mirabilis jalapa* that is devoid of its canonical enzymatic activity, suggesting that the loss of anthocyanins in this species could be due to functional abrogation of ANS. Third, investigations into the *cis*‐regulatory regions of ANS and DFR *in S. oleracea* and *P. americana* have suggested that *cis*‐regulatory modifications could potentially be linked to the observed downregulation of these genes, which is restricted to seed coats (Shimada *et al*., 2004, 2005, 2007; Sakuta *et al*., 2021). However, the impact of *cis*‐modifications remains uncertain, primarily due to the limitations of heterologous promoter‐binding assays (Shimada *et al*., 2007; Sakuta *et al*., 2021). Lastly, in both *Beta vulgaris* and *Portulaca grandiflora*, *trans*‐acting *Promoter of Anthocyanin Pigmentation 1* (PAP1) homologs have been found to lose their ability to interact with canonical bHLH partners in heterologous assays. This loss is suggested to impair the activation of anthocyanin biosynthesis genes *in planta*, thus contributing to the absence of anthocyanins (Hatlestad *et al*., 2014; Sakuta *et al*., 2021).

But while these studies offer valuable insights, their limitations manifest in three ways: (1) they cover a narrow selection of just five species, (2) they lack a consistent evaluation of mechanisms across these species, and (3) they explore only a subset of the components involved in the flavonoid biosynthesis pathway. These limitations lead to gaps in our understanding that warrant further investigation. For instance, the early components of the flavonoid biosynthesis pathway remain largely unexplored, and the evolutionary trajectories of glycosylation enzymes, putative anthocyanin transporters, and a recently identified anthocyanidin synthesis enzyme have not been adequately addressed. Additionally, the evolutionary dynamics of bHLH and WD40 transcription factors, which operate in tandem with the PAP1 transcription factor in an MBW protein complex, have not been investigated. Moreover, varied methodologies and restricted taxon sampling employed in existing research have left it unclear as to whether the mechanisms responsible for the loss of anthocyanins vary across independent transitions to betalain pigmentation. To remedy these gaps, we have capitalized on the recent surge in genomic and transcriptomic resources to construct a comprehensive comparative framework, both gene‐rich and species‐rich. This framework allowed us to reassess the evolutionary fate of the flavonoid biosynthesis pathway in 357 species and 31 families, representing all four proposed transitions to betalain pigmentation.

First, under Hypothesis 1, we scrutinized our dataset for discernable patterns of gene absence in betalain‐pigmented lineages that would indicate wholesale gene loss. Our analyses reveal that most flavonoid synthesis genes are, in fact, conserved within betalain‐pigmented lineages, with the notable patterns of loss of the flavonoid hydroxylase F3′5′H and the wholesale loss of orthologs of TT19, recently identified as a key enzyme in anthocyanidin synthesis (Eichenberger *et al*., 2023). Second, to evaluate Hypothesis 2, we focused on the conserved genes within betalain‐pigmented lineages, probing for shifts in nucleotide substitution patterns that would suggest either loss or alteration of gene function. Our findings indicate a pattern of relaxed purifying selection, as well as reduced diversifying selection for multiple genes, particularly when comparing betalain lineages to their anthocyanin counterparts. Third, to assess Hypothesis 3, we employed combined RNA‐Seq datasets to examine variations in gene expression levels between betalain‐ and anthocyanin‐pigmented families. Our analysis demonstrate diminished expression, especially for late‐stage flavonoid pathway genes in lineages featuring betalain pigmentation. Lastly, for Hypothesis 4, we delved into the MYB‐bHLH‐WD40 (MBW) regulatory complex, examining it for gene loss patterns and site‐specific degeneration of complex‐forming residues specifically within betalain‐pigmented lineages. We observed relaxed selective pressures and gene loss in several constituents of the MBW complex, while residues within the bHLH interaction domain of PAP1 homologs displayed markedly higher divergence and greater diversity in betalain lineages compared with anthocyanin lineages. Overall, our results paint a complex picture supporting repeated anthocyanin loss and underpinned by multiple evolutionary mechanisms, including loss of a key synthetic enzyme and the apparent degeneration of regulatory complexes.

## Materials and Methods

### Data source and processing raw sequences

Some sequence data used in this study were transcriptome and genome assemblies from the One Thousand Plant Transcriptome (1KP) project (Matasci *et al*., 2014) with a larger data sample provided by Caryophyllales‐specific transcriptomic and genomic sampling projects (Walker *et al*., 2018; Pucker *et al*., 2020a). Additional transcriptome assemblies were generated based on publicly available RNA‐Seq datasets of *Halostachys caspica*, *Myosoton aquaticum*, *Oxyria digyna*, *Achyranthes bidentata*, *Dysphania schraderiana*, *Hammada scoparia*, *Hololachna songarica*, and *Gymnocarpos przewalskii* using a previously established protocol (Haak *et al*., 2018). Briefly, this involved trimming with Trimmomatic v.0.39 (Bolger *et al*., 2014) followed by an assembly with Trinity v.2.4 (Grabherr *et al*., 2011) with *k* = 25 and a prediction of peptide sequences (Haak *et al*., 2018). The completeness of the predicted peptides in transcriptome and genome assemblies was evaluated through the presence of well‐conserved Benchmarking Universal Single Copy Orthologs (Buscos) with Busco v.3 (Simão *et al*., 2015), run in protein mode with an *e*‐value cutoff of 1e‐3 and considering at most 10 hits on all predicted peptide sets using the ‘embryophyta odb9’ reference gene set (Zdobnov *et al*., 2017). Genomic or transcriptomic data were retrieved for a total of 357 species: 124 anthocyanin species and 233 betalain species. Of these, we retrieved RNA‐Seq datasets for 110 anthocyanin species and 187 betalain species. Of the RNA‐Seq datasets, the following passed filtering steps (discussed in the ‘Quantifying gene expression’ in the Material and Methods section): 1420 datasets from anthocyanin species and 2374 datasets from betalain species.

### Identification of candidate sequences

To perform a comprehensive analysis of the flavonoid biosynthesis, a thorough annotation of sequences in transcriptome and genome assemblies is required. Annotation is based on sequence similarity to previously characterized sequences. Previously characterized protein sequences for each step in the flavonoid biosynthesis (Pucker *et al*., 2020b), modification, transport, and regulatory pathway including chalcone synthase (*CHS*), chalcone isomerase (*CHI*), flavanone 3‐hydroxylase (*F3H*), flavonoid 3′‐hydroxylase (*F3′H*), flavonoid 3′,5′‐hydroxylase (*F3′5*′*H*), flavonol synthase (*FLS*), flavone synthase (FNS), *DFR*, *LDOX*, leucoanthocyanidin reductase (*LAR*), anthocyanidin reductase (*ANR*), anthocyanidin 3‐*O*‐gucosyltransferase (*A3GT*), anthocyanin 5‐*O*‐gucosyltransferase (*A5GT*), flavonoid 3‐*O*‐glucosyltransferase (*F3GT*), glutathione *S*‐transferase (*AN9/TT19*), proton antiporter (*MATE/TT12*), ATP‐binding cassette protein 1 (*ABC*), autoinhibited H(+)‐ATPase isoform 10 (*AHA10/TT13*), subgroup 6 MYB75 *PAP1*, bHLH proteins EGL1, MYC1 and TT8, and WD40 protein TTG1 were used as baits (search queries) for the identification of candidate sequences with a high degree of similarity to baits. This collection of bait sequences was further extended by identification of orthologous sequences in datasets representing > 120 species of major plant lineages (NCBI and Phytozome datasets) based on a previously described approach (Yang *et al*., 2015). Smith–Waterman alignment‐based searches with Swipe v.2.0.12 (Rognes, 2011) were conducted against each transcriptome or genome assembly, and up to 100 hits per bait with a minimum bit score of 30 were considered in the initial step and manually refined through iterative construction of gene trees with FastTree2 (Price *et al*., 2010) and removal of sequences on long branches likely to represent distantly related or nonhomologous sequences. Next, the extended set of bait sequences was used to further identify candidate sequences in the Caryophyllales following the same iterative approach (Yang *et al*., 2015). Alignments are inferred with Mafft v.7.475 with default auto settings (Katoh & Standley, 2013). Trees are inferred with FastTree2 and manually reviewed for long branches, which were removed. Alignment and tree inference are repeated iteratively until no further long branches are identified.

### Construction of phylogenetic trees

To confirm the phylogenetic identity of candidate sequences and to establish homology relationships, including both orthology and paralogy, we constructed phylogenetic trees for each family of candidate genes. For the construction of gene trees, peptide sequences of outgroup species and Caryophyllales were aligned via Mafft v.7.475 using default auto settings (Katoh & Standley, 2013). Next, the aligned amino acids were substituted with the corresponding codons using pxaa2cdn from Phyx (Brown *et al*., 2017). Alignment columns with occupancy below 10% were removed via Phyx (Brown *et al*., 2017) (pxclsq –p 0.1). RAxML‐NG v.0.9 (Kozlov *et al*., 2019) was used to generate final trees using the GTR + G model and 100 rounds of bootstrapping. Monophyletic or paraphyletic groups of sequences from a single species' transcriptome assemblies could represent true paralogs or isoforms and were reduced to one representative sequence using a publicly available script (Yang & Smith, 2014). Briefly, clusters of monophyletic sequences of a single species were identified and reduced to the single longest transcript in the cleaned alignment. Paraphyletic sequences that are at most one node away from the monophyletic cluster were also masked. Several iterations of tree building and manual cleaning were performed to generate the final gene trees. For example, exceptionally long branches on isolated sequences can sometimes indicate an alignment or annotation issue which escaped initial filtering, where difficult‐to‐explain long branches were recognized, and the alignment was manually examined to understand any issues – sequences which were clearly mis‐annotated on part of their length or otherwise suspiciously misaligned were manually removed. Additional outgroup sequences were included to distinguish between related gene families: stilbene synthases and other polyketide synthases for *CHS*, short‐chain dehydrogenases for DFR (Moummou *et al*., 2012). Sequences of closely related gene families were investigated in a joined alignment and tree to ensure proper assignment of the candidate sequences. F3′H and F3′5′H were investigated together as they are Cytochrome P450 enzymes located in two sister subfamilies CYP75A and CYP75B, respectively. F3H, FLS, and LDOX were analyzed together to clearly separate these closely related 2‐oxoglutarate‐dependent dioxygenase sequences. We used an overlap‐based approach to label duplication nodes in the gene tree, requiring at least two species to overlap between the two daughter clades to map a gene duplication event to a node, and therefore, only detect deeper level gene duplication events represented by at least two species in our taxon sampling.

### Molecular evolution

To assess the signal of altered selection in betalain‐pigmented lineages relative to anthocyanin‐pigmented lineages, we used the branch model approach of Yang (1998), which compare a model with one ratio of nonsynonymous to synonymous substitution rate (dN/dS) for the whole tree to one which partitions the tree into background (anthocyanin) and foreground (betalain) branches, each with their own dN/dS ratio. We reasoned that wholesale loss of function in anthocyanin pathway homologs in betalain‐pigmented lineages would lead to relaxation of purifying selection across the gene and dN/dS at all sites to 1. Because these analyses can be computationally intractable and numerically unstable when the number of parameters exceeds the number of datapoints, we first subsampled each alignment by extracting the clade(s) corresponding to Caryophyllales and then subsampled the resulting tree using a strategy designed to maintain paralogous diversity (Sheehan *et al*., 2020), using the script subsample_by_paralog.py available from https://github.com/NatJWalker‐Hale/alignment_and_tree_tools. The output number of sequences was set to 100 (however, due to differing numbers of paralogues and the requirement to keep at least one sequence per family, the final sample size could differ). Matching sequences were aligned with Guidance2 in codon mode using Mafft (‐‐genafpair ‐‐maxiterate 1000; Katoh & Standley, 2013; Sela *et al*., 2015). Residues with a Guidance2 score lower than 0.6 were masked as Ns, and alignment columns with occupancy below 10% were removed with pxclsq (‐p 0.1). We reasoned that the tree from the full sequence set would likely be more accurate due to the beneficial impact of taxon sampling, so we paired the alignments with the subsampled trees from above. We labelled foreground branches in the trees using the following rules: the subtending branch and all descendant branches in any clade of exclusively betalain‐pigmented taxa, and any tips from betalain‐pigmented taxa, were labelled as foreground. All other branches were considered background. Codon models were fitted using Paml v.4.10.6 (Yang, 2007). We compared the fit of Model 0 (model = 0, NSsites = 0) and the branch model (model = 2, NSsites = 0), using a likelihood ratio test (LRT) assuming a null distribution of the test statistic of chi‐square with one degree of freedom. For PAP1α and PAP1β, because the Caryophyllales clades were very small, we inferred trees from the Guidance2‐masked alignments as above and labelled foreground branches as above. To complement this analysis, we used the RELAX method as implemented in HyPhy v.2.5.53 (Wertheim *et al*., 2015; Kosakovsky Pond *et al*., 2020), which fits a mixture model of sites under strong purifying, nearly neutral, and diversifying selection, and compares foreground to background branches by raising dN/dS to the power of a selective intensity parameter, *K*, which is < 1 under relaxed selection and > 1 under intensified selection. We compared model fit using the LRT of the RELAX null (*K* = 1) vs the alternative with *K* free to vary.

We reasoned those sites substituting because of relaxed selection in MYBs of the MBW complex would show divergent amino acid states and more variability in betalain lineages compared with anthocyanin lineages. We assessed the signal for site‐specific amino acid divergence by calculating the base‐2 Jensen–Shannon Divergence (JSD) between site‐specific amino acid frequencies for sequences from betalain‐pigmented and anthocyanin‐pigmented lineages with script calc_site_specific_divergence_aa_n_aln.py, which is 0 if the two groups have identical site‐specific amino frequencies at the site, and 1 if there is no overlap in state frequencies between the site. We assessed amino acid signals consistent with relaxed selection by calculating the effective number of amino acids (*N*_eff_; Johnson & Wilke, 2020) using the script calc_site_specific_aa_var.py, which scales from 1 to 20 depending on the number of amino acids and their frequencies represented at the site, such that sites with a larger number of different amino acids represented will have a higher *N*_eff_. Both scripts are available from https://github.com/NatJWalker‐Hale/alignment_and_tree_tools. We focused on the region of the PAP sequence inferred to contain the bHLH interacting domain (Zimmermann *et al*., 2004; Hatlestad *et al*., 2014; Sakuta *et al*., 2021), and compared sequences from Caryophyllales betalain‐pigmented taxa to sequences from all anthocyanin‐pigmented taxa, including non‐Caryophyllales outgroups.

### Quantifying gene expression

To assess the relative expression of flavonoid pathway genes and their regulators in betalain‐pigmented vs anthocyanic lineages, we collated publicly available RNA‐Seq data. We collected a comprehensive set of 4071 publicly available RNA‐Seq datasets (i.e. individual sequencing runs) of the Caryophyllales (https://github.com/bpucker/CaryoAnthoBlock). While public RNA‐Seq datasets are a valuable resource, metadata about the experimental settings can be incomplete or inaccurate, for example the classification of DNA sequencing data as RNA‐Seq. Quantification of transcript abundances was performed for each sequencing run individually based on the best available assembly and annotation, as adjudged by gene space completeness assessed by Busco. As UTR annotation or representation in a transcriptome assembly is error‐prone, only coding sequences were used for the quantification of transcript abundances. kallisto v.0.44 was applied with default parameters to quantify read abundance (Bray *et al*., 2016). While Kallisto can automatically determine the necessary settings for paired‐end data sets, some parameters need to be specified for single‐end RNA‐Seq datasets. Because we generally do not know the fragment size in libraries of single‐end RNA‐Seq datasets, an average fragment size of 200 bp with a standard deviation of 100 bp was assumed for all samples. Individual count tables from each dataset were merged to generate one table per species and filtered using customized Python scripts (https://github.com/bpucker/CaryoAnthoBlock). Filtering steps were applied to exclude datasets that are likely not suitable for quantitative analyses. Substantial amount of reads in an RNA‐Seq experiment belong to a small number of highly abundant transcripts. Assessing the distribution of transcript abundances in an individual sample allowed the identification and removal of normalized libraries which are characterized by the depletion of abundant transcripts and other artifacts which would not be suitable for quantitative analyses. The proportion of expression assigned to the 100 most abundant transcripts (top100) was determined for all datasets. Cutoffs were identified based on the distribution of these values: Only datasets with > 10% and < 80% of the total transcript per million (TPM) assigned to the top100 transcripts were subjected to down‐stream analyses. Datasets failing these filter criteria would not be suitable for a quantitative analysis. 3933 RNA‐Seq datasets belonging to 301 species passed these filters. Where possible, only paired‐end datasets were considered, because these reads can be assigned to similar transcripts with higher confidence.

Gene expression was compared between anthocyanin‐pigmented and betalain‐pigmented lineages for all genes in the flavonoid biosynthesis. Because the number of data sets is highly variable between species, all species are represented by the mean of expression values of all valid samples. This reduction to one data point per species avoids an overrepresentation of species with many available data sets. The sum of the transcript abundances (TPMs) of all isoforms of a given flavonoid biosynthesis gene were added up per RNA‐Seq sample (Supporting Information Fig. S1). Isoforms are all sequences that were assigned to the same function in the pathway through steps described above (i.e. all sequences included in each homolog tree, including paralogues). The combination of large numbers of datasets generated for different tissues under various conditions results in a high level of noise. Thus, only strong systemic biological signal should emerge from the broad‐scale comparative analysis, with precise quantifications of gene expression differences among different lineages being infeasible. The average value representing each species comprises a species‐specific number of samples that have different levels of variability resulting from multiple sources, and we therefore did not attempt to model this intraspecies variability. As each species is represented with a single average expression value per gene in the comparison between pigmentation groups, the most abundant tissue type was identified for each species to approximate the tissue representing the majority signal. The available metadata were compared between anthocyanin‐pigmented and betalain‐pigmented groups to preclude the possibility that differences resulted from tissue sampling alone (Table S1).

## Results

### Hypothesis 1: wholesale gene loss in betalain‐pigmented lineages

**Fig. 3:**
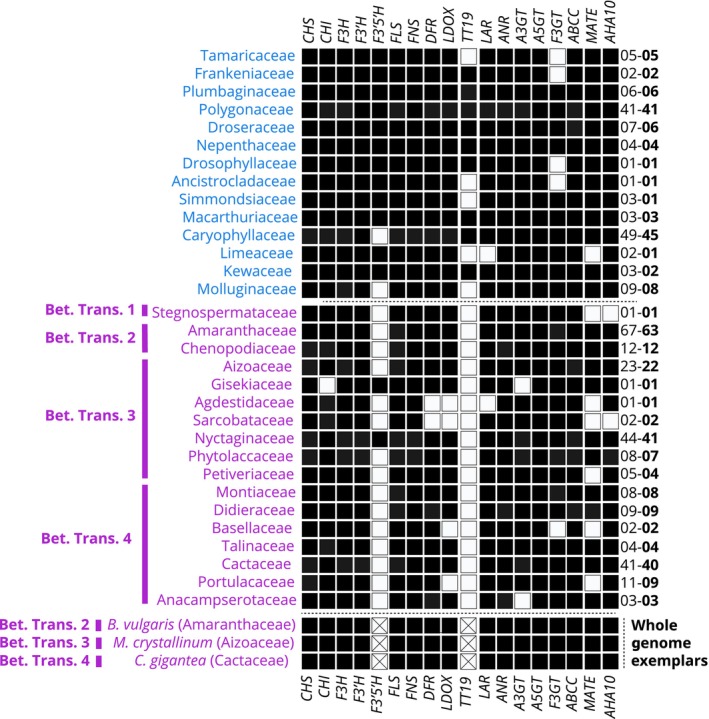
Detection of flavonoid biosynthesis genes in 357 Caryophyllales species summarized at the family level. Families are sorted by pigmentation state into anthocyanin‐ and betalain‐pigmented (blue = anthocyanin, purple = betalain) to highlight the consistent differences between pigment types. Generally, most genes of the flavonoid biosynthesis are present in most families. Only *F3′5′H* and *TT19* are consistently missing from betalain‐producing families. Species with exceptionally well‐annotated contiguous genome sequences that represent the three betalain origins were included at the bottom in italics to add additional support to the pattern. *CHS* (naringenin‐chalcone synthase), *CHI* (chalcone isomerase), *FNS* (flavone synthase), *FLS* (flavonol synthase), *F3H* (flavanone 3‐hydroxylase), *F3′H* (flavonoid 3′‐hydroxylase), *F3′5′H* (flavonoid 3′,5′‐hydroxylase), *DFR* (dihydroflavonol 4‐reductase), *LDOX* (leucoanthocyanidin dioxygenase), *LAR* (leucoanthocyanidin reductase), *ANR* (anthocyanidin reductase), *A3GT* (anthocyanidin 3‐*O*‐glucosyltransferase), *A5GT* (anthocyanin 5‐*O*‐glucosyltransferase), *F3GT* (flavonoid 3‐*O*‐glucosyltransferase), *TT19* (glutathione *S*‐transferase), *MATE* (proton antiporter), *ABC* (ATP binding cassette protein 1), and *AHA10* (autoinhibited H(+)‐ATPase isoform 10). Black = presence in at least one transcriptome or genome assembly in the family, white = not detected in transcriptome and genome assemblies, white with a cross = absence (unable to detect in highly contiguous genome sequence). The number on the right‐hand side indicates the number of transcriptome and genome assemblies sampled (plain text) and the number of species (bold). Below the dotted line are three highly contiguous genome assemblies representing 3/4 of the putative betalain transitions.

We searched for 18 genes in the flavonoid pathway within our transcriptome and genome sequence assemblies and generated phylogenetic trees (Fig. S2) to explore relationships, gene loss, and gene duplication (Fig. S3): *CHS*, *CHI*, *F3H*, *F3′H*, *F3′5′H*, *FLS*, *DFR*, *LDOX*, *LAR*, *ANR*, *A3GT*, *A5GT*, *F3GT*, *AN9*, *MATE*, *AHA10*, and *ABCC*. The bulk of datasets used in this analysis are transcriptomic in origin and can only offer proof of gene presence, as apparent gene absence may simply be due to lack of expression. However, coupled with annotated genome assemblies representing three of the inferred transitions to betalain pigmentation (*B. vulgaris*, *Mesembryanthemum crystallinum*, and *Carnegiea gigantea*), the combined genomic and transcriptomic datasets are informative with respect to the broad‐scale patterns of low gene expression and/or loss (Fig. 3), for a heatmap version (see Fig. S4).

In Fig. 3, we focused on evidence for deeper level gene loss with the gene data summarized at the level of family, in line with data on pigment status which is historically also summarized to family level. Because these patterns are represented at the family level, there may be sub‐familial or species‐specific patterns of loss that may be significant, but are not captured in this representation. In some anthocyanin‐pigmented families, we detected occasional sporadic gene absence without apparent phylogenetic pattern for: *F3′5′H*, *LAR*, *F3GT*, *AN9*, and *MATE*. Except for F3′5′H loss in Caryophyllaceae (which has high data coverage), these apparent gene absences in anthocyanic taxa usually appeared in lineages with very little transcriptomic coverage (median of 2 transcriptome assemblies) and therefore higher probability of stochastic lack of detection. In general, most flavonoid biosynthesis, decoration, and transport‐associated genes are maintained and expressed in betalain‐pigmented families. But in some betalain‐producing families that lack whole genome data, we were unable to find transcriptomic evidence for the following genes: *F3GT*, *MATE*, and *AHA10* in Stegnospermataceae, *CHI*, *F3H*, and *F3′H* in Gisekiaceae; *CHI*, *DFR*, *ANS*, *LAR*, and *ANR* in Agdestidaceae; *CHI*, *F3H*, *DFR* and *ANS* in Sarcobataceae; *ANS* and *LAR* in Basellaceae; *ANS*, *LAR* and *ANR* in Portulacaceae. However, Stegnospermataceae, Gisekiaceae, Agdestidaceae, and Sarcobataceae are all monotypic families, comprising only a single species, and represented by a one or two transcriptome assemblies in our analyses, again representing a higher probability of lack of detection (Fig. 3).

**Fig. 4:**
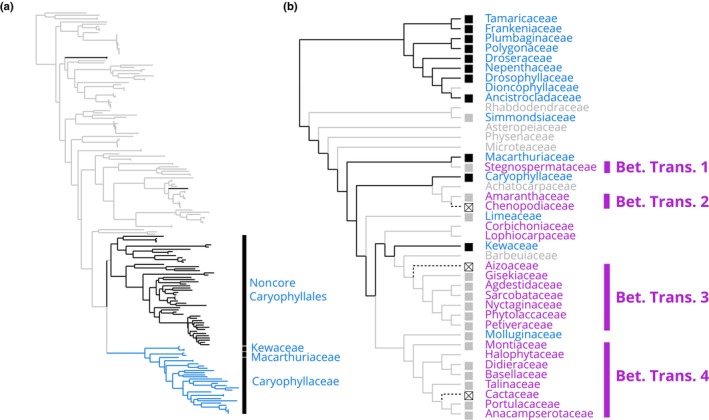
Loss of *TT19* orthologs in betalain‐pigmented lineages. (a) A phylogenetic analysis revealed the presence of *TT19* homologs in most anthocyanin‐pigmented Caryophyllales species, but the absence from all betalain‐pigmented species in 31 families sampled (gray = non‐Caryophyllales outgroups, black = noncore anthocyanic Caryophyllales, blue = anthocyanic core Caryophylales). (b) Parsimony‐based reconstruction of *TT19* loss assuming losses are irreversible, and with the conservative assumption that absence of a gene from the transcriptome is not proof of absence. Black lines = presence, gray lines = ambiguous, dotted lines = loss, blue = anthocyanin, purple = betalain, black box = *TT19* gene detected, gray box = no *TT19* detected, crossed box = *TT19* not detected in highly contiguous genome sequence, no box = missing data.

Putative stochastic absences aside, two stronger patterns of gene absence emerge in relation to betalain‐pigmentation lineages. First, we found no evidence for the presence of F3′5′H in 15 out of 17 betalain‐pigmented families including in genome assemblies from *B. vulgaris*, *M. crystallinum*, and *C. gigantea*. Second, we recovered a striking pattern of repeated absence from transcriptome and genome assemblies for the protein TT19 in betalain‐pigmented lineages that could be explained by gene loss (Fig. 3). In the phylogenetic tree of TT19 orthologs (Fig. 4a), we recovered numerous sequences from anthocyanic noncore Caryophyllales species and core Caryophyllales anthocyanin‐pigmented Caryophyllaceae, Macarthuriaceae, and Kewaceae. Importantly, only anthocyanin‐pigmented species are represented in this tree, and no sequences were detected from a betalain‐pigmented species. These species represent independent betalain‐pigmented lineages, and our phylogenetic reconstruction supports the fact that *TT19* orthologs have been separately and completely lost in multiple betalain lineages (Fig. 4b). The probability of missing *TT19* by chance in all betalain‐pigmented families (0/17) if the proportion of absence in anthocyanin‐pigmented families (5/14) represents the probability of failing to detect *TT19* when it is present, would be below 0.001 (binomial probability). This conservative estimation does not account for the better representation of betalain‐pigmented species within our datasets.

### Hypothesis 2: loss or change in function in flavonoid pathway genes

**Fig. 5:**
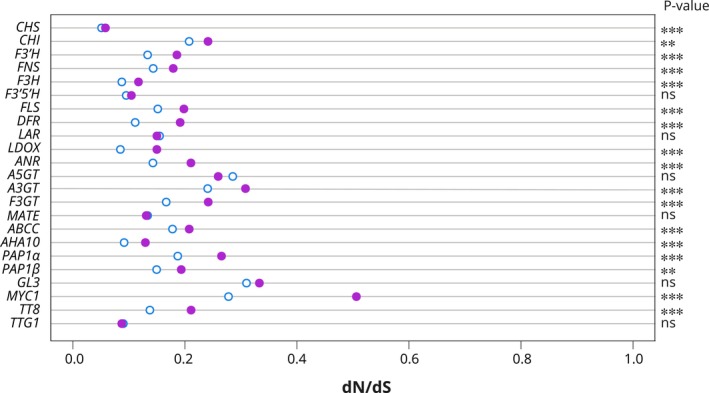
Signature of selection in flavonoid pathway genes in betalain vs anthocyanin lineages. Ratio of nonsynonymous to synonymous substitutions between 17 pathway genes (and 6 regulator genes – see later the Results section for description of these) from anthocyanin‐pigmented taxa (white circle with blue boundary) and genes from betalain‐pigmented taxa (filled purple circles; excluding TT19 as no recovered from betalain lineages) averaged over all codons in the alignment. *CHS* (naringenin‐chalcone synthase), *CHI* (chalcone isomerase), *FNS* (flavone synthase), *FLS* (flavonol synthase), *F3H* (flavanone 3‐hydroxylase), *F3′H* (flavonoid 3′‐hydroxylase), *F3′5′H* (flavonoid 3′,5′‐hydroxylase), *DFR* (dihydroflavonol 4‐reductase), *LDOX* (leucoanthocyanidin dioxygenase), *LAR* (leucoanthocyanidin reductase), and *ANR* (anthocyanidin reductase), *A3GT* (anthocyanidin 3‐*O*‐glucosyltransferase), *A5GT* (anthocyanin 5‐*O*‐glucosyltransferase), *F3GT* (flavonoid 3‐*O*‐glucosyltransferase), *MATE* (proton antiporter), *ABC* (ATP binding cassette protein 1), and *AHA10* (autoinhibited H(+)‐ATPase isoform 10), *PAP1α* and *PAP1β* (subgroup 6 MYB transcription factors), *GL3*, *MYC1*, and *TT8* (bHLH transcription factors) and *TTG1* (WD40 protein). Several genes in betalain‐pigmented lineages show slightly elevated but significant dN/dS indicating slightly relaxed selection relative to anthocyanic lineages but still < 1, consistent with purifying selection. Likelihood ratio test (LRT) *P*‐values (see the Materials and Methods section): ns, not significant; **, *P* < 0.01; ***, *P* < 0.001.

We searched for molecular evolutionary signatures of relaxed selection consistent with loss of function in structural and transport genes using branch codon models which allow for a different ratio of synonymous and nonsynonymous substitutions between genes from anthocyanin‐pigmented and genes from betalain‐pigmented taxa, averaged over all codons in the alignment. Many genes in the pathway showed a significantly better fit of the two‐ratio model over the null model, and the dN/dS in betalain‐pigmented lineages tended to be elevated over anthocyanin‐pigmented lineages, but still < 1 so consistent with purifying selection (Table S2; Fig. 5). In these cases, the difference in dN/dS between anthocyanin‐ and betalain‐pigmented lineages was small, with the greatest increase (*c*. 0.23) observed for *MYC1* (Fig. 5). The two‐ratio model did not offer significant improvement in fit for *F3′5′H*, *LAR*, *A5GT*, *MATE*, *GL3*, and *TTG1*, and consistently, the inferred difference in dN/dS between anthocyanin‐ and betalain‐pigmented lineages for these genes was very small. RELAX results were largely consistent, inferring significant relaxation of selection for all genes except *F3′5′H*, *LAR*, *A3GT*, *MATE*, *PAP1β*, *GL3*, and *TTG1* (Table S3; Fig. S5). *A5GT* was inferred to be under intensified selection, consistent with the reduced average dN/dS in betalain lineages compared with anthocyanin lineages inferred from the branch model.

### Hypothesis 3: reduced expression of flavonoid pathway genes in betalain‐pigmented lineages

**Fig. 6:**
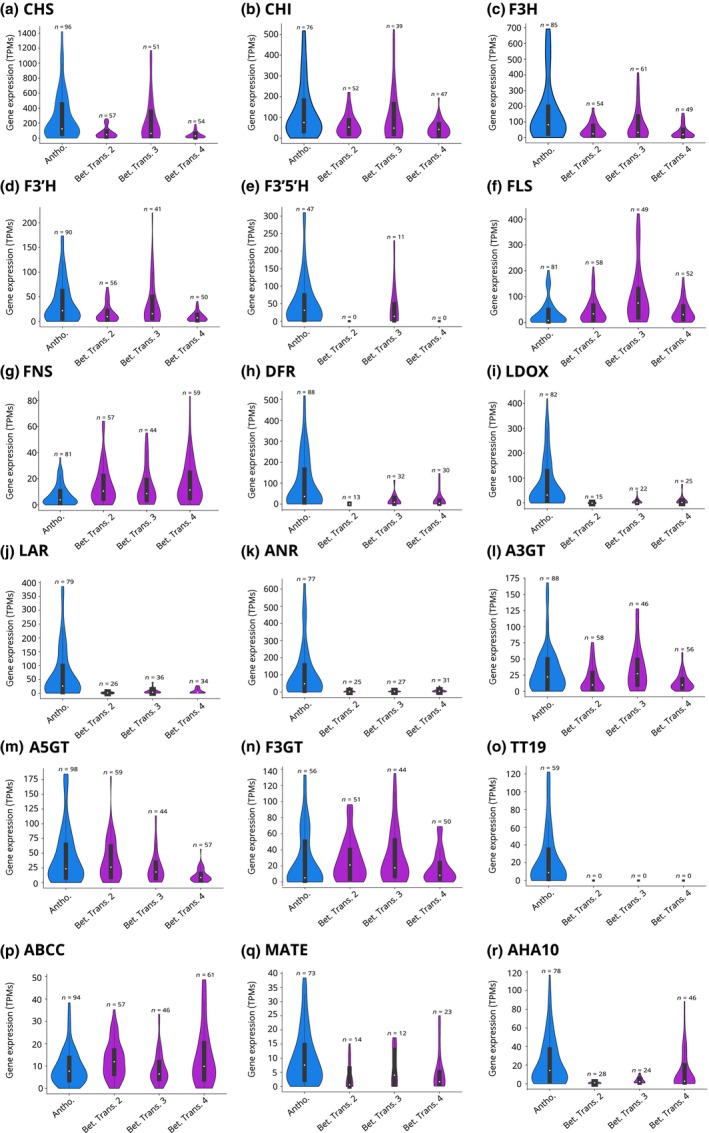
Comparative analysis of flavonoid biosynthesis gene expression in the Caryophyllales. The gene expression in anthocyanin‐pigmented species (blue) is compared with the gene expression in species of three betalain transitions (pink) (see Fig. 1b for illustration of three origins). (a) *CHS* (naringenin‐chalcone synthase), (b) *CHI* (chalcone isomerase), (c) *F3H* (flavanone 3‐hydroxylase), (d) *F3′H* (flavonoid 3′‐hydroxylase), (e) *F3′5′H* (flavonoid 3′5′‐hydroxylase), (f) *FLS* (flavonol synthase), (g) *FNS* (flavone synthase), (h) *DFR* (dihydroflavonol 4‐reductase), (i) *LDOX* (leucoanthocyanidin dioxygenase), (j) *LAR* (leucoanthocyanidin reductase), (k) *ANR* (anthocyanidin reductase), (l) *A3GT* (anthocyanidin 3‐*O*‐gucosyltransferase), (m) *A5GT* (anthocyanidin 5‐*O*‐glucosyltransferase), (n) *F3GT* (flavonoid 3‐*O*‐glycosyltransferase), (o) *TT19* (glutathione *S*‐transferase), (p) *ABCC* (ATP binding cassette protein 1), (q) *MATE* (proton antiporter), and (r) *AHA10* (autoinhibited H(+)‐ATPase isoform 10). Blue = anthocyanin‐pigmented lineages, purple = betalain‐pigmented lineages. Boxplots within violin plots show median (white dot), interquartile range (black box), and maximum and minimum values excluding outliers (whiskers).

We observed a generally reduced transcript abundance in most genes in the flavonoid biosynthesis pathway in betalain‐pigmented vs anthocyanin‐pigmented species (Fig. 6). This observation was very common, but the differences are far more dramatic for some genes than others. This pattern is apparent for some early‐acting components (*CHS*, *CHI*, *F3H*, *F3′H*, and *F3′5′H*) but is especially pronounced for the late‐acting components (*DFR*, *LDOX*, *LAR*, *ANR*, *MATE*, and *AHA10*). This phenomenon was clearly visible in data representing the three putative betalain origins that were sampled. Although expression of later‐acting flavonoid biosynthesis genes leading to anthocyanins and proanthocyanidins are highly reduced in betalain‐pigmented species, several genes acting in other branches of the flavonoid biosynthesis show little reduction. For example, the flavonol biosynthesis gene *FLS* shows almost no difference between anthocyanic and betalain‐pigmented lineages. Although *CHS* transcript was observed at substantially lower abundance in betalain‐pigmented species, its abundance is still relatively high compared with other genes in the pathway, implying a substantial production of the key flavonoid substrate naringenin chalcone in betalain‐pigmented lineages.

### Hypothesis 4: loss and/or degeneration of the MBW complex in betalain‐pigmented lineages

Given the observed reduction in gene expression, especially in late‐acting components in the flavonoid pathways, we then examined the evolutionary fate of the MYB, bHLH, and WD40 transcription factors which together are known to regulate at least *DFR* and *LDOX* expression in the production of anthocyanins. Specifically, we searched for the following five genes in the flavonoid pathway within our transcriptome and genome sequence assemblies and generated phylogenetic trees (Fig. S6) to explore patterns of loss: *PAP1*, *Glabrous3* (*GL3*), *Transparent Testa 8* (*TT8*), *Myelocytomatosis Oncogene 1* (*MYC1*), and *Transparent Testa Glabra 1* (*TTG1*).

**Fig. 7:**
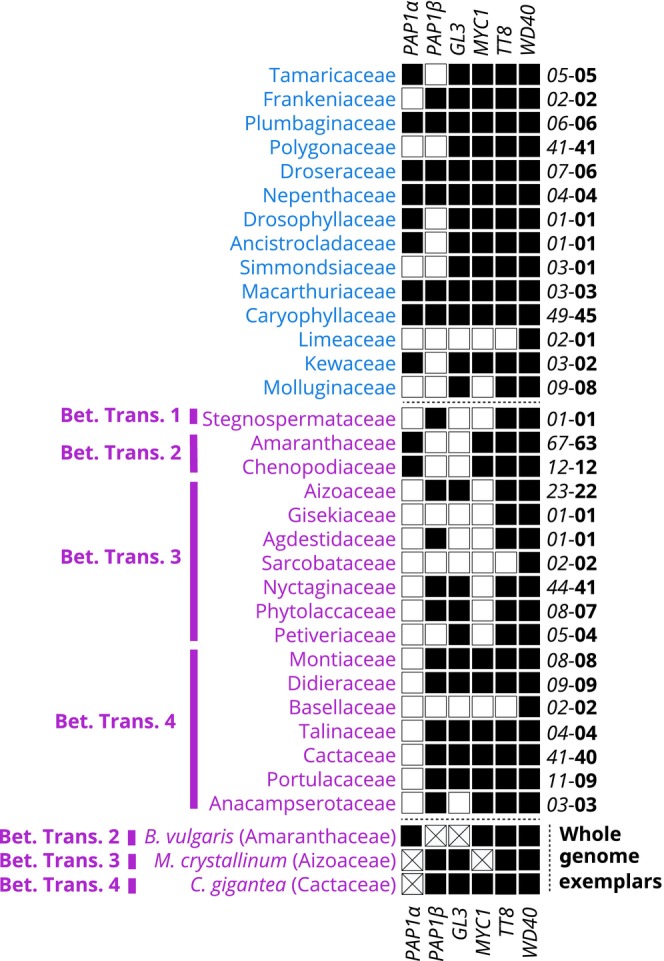
Detection of components of the anthocyanin MBW complex in 357 Caryophyllales species summarized at the family level. Families are sorted by pigmentation state into anthocyanin‐ and betalain‐pigmented (blue = anthocyanin, purple = betalain) to highlight the consistent differences between pigment types. The detection of homologs of five components is reported: WD40 TTG1, bHLHs TT8, MYC1, and GL3, and MYB PAP1s (PAP1α and PAP1β). Black = presence in at least one transcriptome or genome assembly in the family, white = not detected in transcriptome or genome assemblies, white with a cross = absence unable to detect in most contiguous genome sequence. Number on the right‐hand side indicates the number of transcriptome and genome assemblies sampled (italics) and the number of species (bold). Below the dotted line are three highly contiguous genome assemblies representing 3/4 of the putative betalain transitions.

The WD40 TTG1 lineage is universally recovered in all betalain and anthocyanin families and shows no evidence of gene loss. Likewise, of the three bHLH lineages known to interact in the MBW complex, the TT8 bHLH lineage, which is implicated in proanthocyanidin pigmentation, is substantially present across all betalain and anthocyanin families (Fig. 7), aside from occasional absences in three families with more limited transcriptome data (Limeacee, Sarcobataceae, and Basellaceae). However, the other two bHLH lineages – the MYC1 and GL3/EGL3 lineages – show more evidence of absence from transcriptomes and corresponding whole genome sequences. MYC1 homologs were present in 12/14 anthocyanin‐pigmented families but only present in 8/17 betalain families. MYC1 homologs were not recovered from any transcriptomes emanating from Betalain Transition 3, consonant with its loss in the *M. crystallinum* genome (Fig. 7). The GL3 lineage is recovered in 13/14 anthocyanic families but only present in 9/17 betalain‐pigmented families and is not recovered from any transcriptomes emanating from Betalain Transition 2, consonant with its loss in *B. vulgaris* (Fig. 7).

**Fig. 8:**
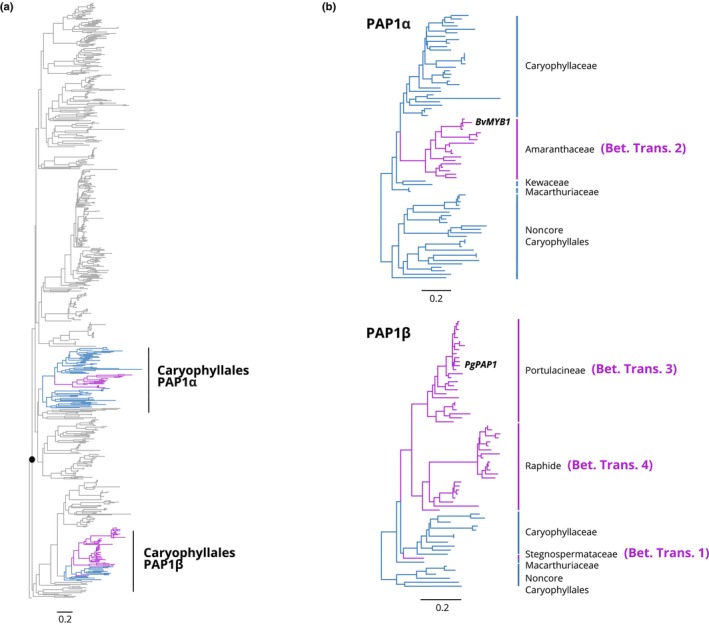
Phylogeny of the *Promoter of Anthocyanin Pigmentation 1* (PAP1) MYB lineage. (a) Phylogeny of the PAP1 lineage across eudicots revealing the existence of a deep duplication (marked with solid black circle) that gives rise to PAP1α and PAP1β clades, both of which are represented in Caryophyllales. (b) Phylogenies of the two paralogous PAP1 lineages (PAP1α and PAP1β), illustrating their differential and reciprocal patterns of loss and retention in the four transitions to betalains. Betalain branches are purple and anthocyanin branches are blue. Scale bars indicate the expected number of substitutions per site.

The PAP1 lineage shows a complex pattern of duplication across the eudicots, resulting in two paralogous lineages of PAP1 in Caryophyllales, which stem from a duplication preceding the origin of Caryophyllales, and which we have termed PAP1α and PAP1β (Fig. 8a). The PAP1α lineage is recovered in 9/14 anthocyanic families, but only present in 2/17 betalain families, and only recovered from transcriptome assemblies derived from Amaranthaceae and Chenopodiaceae, consistent with its presence in the *B. vulgaris* genome (Fig. 7). PAP1α was undetected from any transcriptome assemblies derived from Betalain Transitions 1, 3 and 4, consonant with their loss in the *M. crystallinum* and *C. gigantea* genomes. The PAP1β lineage, on the contrary, is only recovered from 6/14 anthocyanic families, and present in 11/17 betalain families. PAP1β homologs are not recovered from any transcriptome assemblies emanating from Betalain Transition 2, consonant with its loss in the *B. vulgaris* genome. Conversely, PAP1β homologs are only detected in families arising from Betalain Transitions 1, 3, and 4, consistent with their presence in the *M. crystallinum* and *C. gigantea* genomes. In summary, PAP1α and PAP1β exhibit reciprocal patterns of retention and loss that are a mirror image of each other (Fig. 8b).

**Fig. 9:**
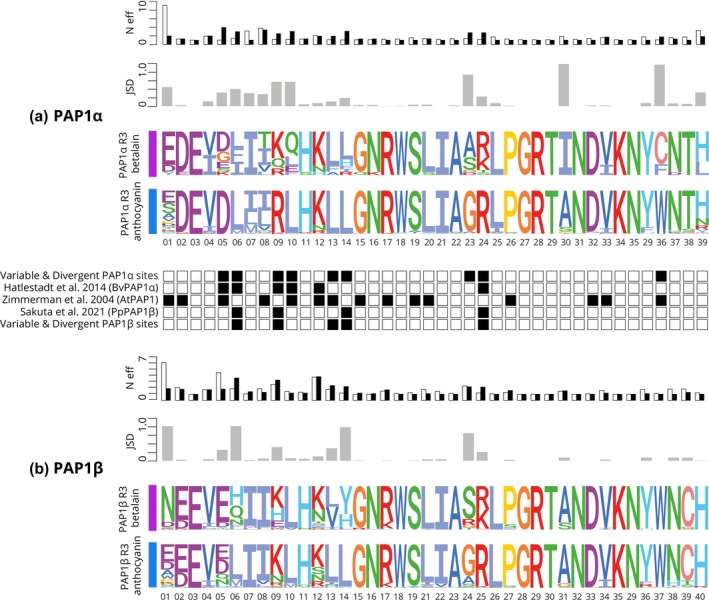
Comparison of substitution patterns occurring in the R3 interacting domain of PAP1α and PAP1β homologs in betalain vs anthocyanic lineages. Residue‐level analysis of the 40‐amino acid R3 interacting domain from the study by Zimmermann *et al*. (2004). The figure presents the data for: (a) PAP1α and (b) PAP1β. Both proteins, PAP1α and PAP1β, feature: *N*_eff_ (effective number of amino acids) plots, with anthocyanin in white and betalains in black; JSD (Jensen–Shannon Divergence) plots; and logo plots for both anthocyanic and betalain‐pigmented lineages. Central to the figure is a black‐and‐white matrix, highlighting residues (represented by back squares) that are crucial for MYB‐bHLH interactions. Their relevance has been backed by three distinct studies: Zimmermann *et al*. (2004) for AtPAP1, Hatlestad *et al*. (2014) for PAP1α, and Sakuta *et al*. (2021) for PAP1β. Furthermore, residues shown as consistently divergent and notably variable between betalain and anthocyanic lineages are also marked with black squares. These were deduced from the *N*_eff_, JSD, and logo analyses.

We then examined both PAP1α and PAP1β lineages, looking at the MYB‐bHLH interacting domain (N‐terminal, R3 repeat), which is essential for canonical MBW complex formation. Zimmermann *et al*. (2004) identified 15 residues within the R3 repeat that are essential in mediating the interaction of AtPAP75 and AtbHLH proteins (Fig. 9, positions: 1, 2, 5, 8, 10, 12, 13, 15, 17, 19, 20, 26, 32, 33, and 36). Hatlestad *et al*. (2014) analyzed PAP1α representative BvMYB1 from *B. vulgaris* and identified six residues which when mutagenized could restore the lost MYB‐bHLH interaction (Fig. 9, positions: 5, 6, 9, 10, 12, and 24). Sakuta *et al*. (2021) analyzed the PAP1β representative PgPAP1 and identified four residues in the interacting domain which when mutagenized could restore canonical PAP1 function (Fig. 9, positions: 5, 9, 13, 14, and 24). Combining existing functional data from site‐specific mutagenesis studies, in the context of our new phylogenetic resolution, the PAP1α and PAP1β lineages show convergence in degenerated interacting sites, at positions 6, 9, and 24. Applying bioinformatic approaches, we found further evidence for clear divergence (JSD) in the identity of residues found in betalain vs anthocyanin PAP1s, coupled with greater site‐specific variation (Neff) in betalain vs anthocyanin PAP1s. For PAP1α, sites showing elevated divergence and variation include 5, 6, 9, 10, 13, 14, 23, 24, and 36 (Fig. 9). For PAP1β, sites showing elevated divergence and variation include 6, 9, 13, 14, and 24. Based on bioinformatic signals, therefore, PAP1α and PAP1β showed additional patterns of convergence at sites 13 and 14. Both PAP1 lineages, MYC1, and TT8 all showed higher elevated dN/dS in betalain‐pigmented lineages compared with anthocyanin‐pigmented lineages, consistent with some relaxation of purifying selection, while GL3 and TTG1 did not (Fig. 5; Table S2).

## Discussion

### Significant patterns of loss in *TT19* and *F3′5′H* gene lineages within betalain‐pigmented families

Previously, the TT19 family of glutathione *S*‐transferases, including *AN9* gene in *Petunia hybrida* and its *TT19* ortholog in *Arabidopsis thaliana*, was primarily linked with anthocyanin transport and stabilization (Mueller *et al*., 2000). The prevalent model suggested that TT19 orthologs play a key role in shuttling anthocyanins from the endoplasmic reticulum to the tonoplast for further processing (Sun *et al*., 2012). However, a very recent study has revealed a different major function for the *TT19* orthologs. Instead of transport, TT19 enzymes are now suggested to predominantly catalyze the dehydration of flavan‐3,3,4‐triol, a product of LDOX, to produce cyanidin (Eichenberger *et al*., 2023). This casts TT19 as a critical enzyme in the late stages of anthocyanin formation. This discovery adds significance to our finding of complete absence of *TT19* orthologs in transcriptome assemblies and annotated genomes of betalain‐pigmented species, despite their detectable presence in three anthocyanin lineages within the core Caryophyllales. Notably, these orthologs are retained in Kewaceae, Caryophyllaceae, and Macarthuriaceae, logically as a plesiomophic state from a common ancestor. The absence in betalain species then implies that *TT19* orthologs have been lost repeatedly in different betalain origins (Fig. 4b). In contrast to earlier research that relied on the presence of *DFR* and *LDOX* genes as late‐stage markers for a functioning anthocyanin synthesis pathway, our findings indicate that anthocyanin synthesis has in fact been repeatedly disrupted through the loss of the enzyme crucial for converting flavan‐3,3,4‐triol to anthocyanidin.

The repeated loss of *TT19* orthologs impacts on our understanding of the directionality in pigment trait evolution. Previously, the maintenance but restricted expression of flavonoid synthesis genes, *LDOX* and *DFR*, in the proanthocyanidin‐pigmented seed coats of betalain‐pigmented species was inferred to give a clear evolutionary mechanism for multiple reversals back to anthocyanin pigmentation from a betalain ancestor (Fig. 1a), that is restoring broader expression patterns of *LDOX* and *DFR* could restore anthocyanin biosynthesis, assuming presence of all other components of the pathway. However, recent research by Sakuta *et al*. (2021) complicates this narrative. Their work shows that anthocyanin pigmentation in betalain‐pigmented *A. myriostigma* can only be reinstated through the heterologous expression of *DFR* and *LDOX* when it is accompanied by the heterologous expression of *PhAN9*, the *TT19* ortholog from *P. hybrida*. Our discovery of the repeated loss of *TT19* orthologs in betalain‐pigmented lineages supports the findings of Sakuta *et al*. (2021) and suggests a pattern of recurrent loss of anthocyanins in core Caryophyllales, consistent with the hypothesis of repeated specialization to betalain pigmentation (Sheehan *et al*., 2020).

The enzymes F3′H and F3′5′H play pivotal roles in the flavonoid biosynthesis pathway, specifically at branch points where they convert dihydrokaempferol to either dihydroquercetin or dihydromyricetin, respectively. Both are Cytochrome P450 enzymes, with F3′H belonging to the CYP75B subfamily and F3′5′H to the CYP75A subfamily (Yonekura‐Sakakibara *et al*., 2019). In our study, F3′H was universally present across all examined families (23/23 families), while F3′5′H from the CYP75A lineage was conspicuously absent in from most core Caryophyllales lineages (17/23 families), including almost all betalain‐pigmented lineages (15/17 families). This absence was further corroborated by the three highly contiguous genomes (Fig. 3). Dihydromyricetin, the product of *F3′5′H* activity, can be converted to myricetin‐derived flavonols, and consistent with the loss of F3′5′H orthologs, Caryophyllales predominantly produce quercetin‐type flavonoids via F3′H, rather than the myricetin‐type flavonoids, which are dependent on F3′5′H, and are notably rare (Iwashina, 2015). Dihydromyricetin, produced by F3′5′H activity, also serves as a critical precursor for the blue anthocyanin delphinin. Prior studies have documented the rapid pseudogenization and deletion of F3′5′H in species transitioning from delphinin‐based blue to red‐colored flowers, highlighting its evolutionary significance for blue anthocyanin production in flowers (Smith & Rausher, 2011; Wessinger & Rausher, 2014; Ho & Smith, 2016). Given the widespread loss or downregulation of F3′5′H, it is notable that blue flowers are completely absent in all betalain‐pigmented species, and extremely rare even among anthocyanic Caryophyllaceae species. One interpretation might be that the rare occurrence of blue flowers has led to reduced evolutionary pressure to maintain the F3′5′H enzyme. This, in turn, has a cascading effect, resulting in not only a lack of dihydromyricetin‐derived delphinidin but also a paucity of other myricetin‐derived flavonoids. However, the presence of F3′5′H in some betalain lineages suggests that its loss is not an early, singular event but occurs multiple times toward the tips of the phylogeny. In general, widespread loss of F3′5′H adds a layer of complexity to our understanding of flavonoid biosynthesis and its evolutionary trajectory within Caryophyllales.

### Little evidence for significant altered or loss of function in maintained flavonoid biosynthesis genes within betalain‐pigmented species

Recent work proposed that a truncation in the anthocyanin biosynthesis gene LDOX could account for the absence of anthocyanins in the betalain‐pigmented species *M. jalapa* (Polturak *et al*., 2018). Although all genes for anthocyanin biosynthesis are expressed in *M. jalapa* flowers, no anthocyanins appear, a phenomenon attributed to a truncated form of MjANS (aka LDOX) that failed to complement an *A. thaliana ans/ldox* mutant (Polturak *et al*., 2018). However, contrary to this, our findings indicate the existence of an *LDOX* gene duplication within the Nyctaginaceae family, resulting in two distinct clades. One of these clades includes the truncated MjANS previously detected in *M. jalapa*, but both clades contain full‐length ANS versions as well (Figs S3, S7). *M. jalapa* itself has both truncated and full‐length ANS variants. Given this, we argue that ANS functional loss is probably not the reason for the total absence of anthocyanins in *M. jalapa*. More broadly, our data shows that most betalain‐pigmented species possess a full‐length *LDOX* gene, with no clear genomic evidence supporting a widespread loss of LDOX functionality through frame‐shifting mutations or significant indels.

Looking beyond *LDOX*, we used codon models to explore the signal of relaxed selection indicative of loss of function in other genes in the pathway. We found that branches derived from betalain‐pigmented lineages generally showed elevated dN/dS compared with branches from anthocyanin‐pigmented lineages, when averaging over all codons. The increase in dN/dS we observe could be consistent with relaxed purifying selection in betalain‐pigmented lineages. However, the observed differences are small, with none of the values were above one, indicating that purifying selection is still occurring, and total loss of function is unlikely. Because these branch models average over codons, it is difficult to say exactly from where this signal is derived. For example, the increase in dN/dS could also be explained by a small number of sites experiencing positive selection in foreground branches. Because many of the genes we examined experienced gene duplications in Caryophyllales, and we have not discriminated between paralogs, it is possible that bursts of amino acid substitution associated with neo‐ or subfunctionalization in foreground branches could mislead these tests. On the contrary, RELAX analyses suggested that diversifying selection was frequently also reduced in betalain‐pigmented lineages, but most sites were still inferred to be evolving under strong purifying selection, which likewise suggests that wholesale loss of function in these loci is unlikely.

In general, evidence would also seem to suggest that widescale loss of function is unlikely. Shimada *et al*. (2004, 2005) characterized *LDOX* and *DFR* from *S. oleracea* (Amaranthaceae s. l.) and *P. americana* (Phytolaccaceae) and showed that the genes maintained their canonical function when expressed in *Escherichia coli*. This is in line with expectations based on the widescale maintenance of these homologs in the genomes of betalain‐pigmented taxa. Furthermore, because Caryophyllales still produce proanthocyanidins in the seed, these functions are still required. Similarly, some Caryophyllales still produce most other types of flavonoids (Iwashina, 2015), and it seems likely the functional copies of most of the pathway genes are required to produce these compounds. Therefore, despite the potential signal of marginal relaxed selection, we assess loss or change in gene function across the pathway to be very unlikely, outside the wholesale gene loss previously discussed.

### Numerous flavonoid biosynthesis genes show reduced expression in betalain‐pigmented lineages

Before delving into the discussion of our comparative expression analyses, it is necessary to acknowledge the constraints of our study. First, the RNA‐Seq data sets we utilized were not generated with our specific research objectives and analytical approaches in mind, a common limitation in bioinformatic reanalysis. However, the broad range of species and tissue types represented in these publicly available datasets surpasses what could realistically be collected in a single study. Despite this benefit, inconsistencies exist, including varying numbers of RNA‐Seq datasets across species and uneven sampling across tissues, developmental stages, and stress conditions. Moreover, the lack of corresponding metabolite data precludes the correlation of gene expression patterns with target flavonoids. Complicating matters further, recurrent gene duplication events (Fig. S3) necessitated the integration of expression values across multiple paralogs, many of which were identified for the first time in this study. It is reassuring, however, that the macroevolutionary trends we report appear to be devoid of systematic bias as certain genes like *FLS* deviate from patterns observed in other flavonoid biosynthesis genes. While providing valuable qualitative insights, these analyses serve as a preliminary global examination of flavonoid pathway gene expression and should be interpreted cautiously.

From our analysis, we note a generally reduced expression of most genes involved in the anthocyanin pathway in betalain‐pigmented species compared with anthocyanin‐pigmented counterparts. This reduction is evident across three evolutionary transitions for which sufficient RNA‐Seq data was available. While the expression of early‐acting components such as *CHS*, *CHI*, *F3H*, *F3′H*, and *F3′5′H* is modestly reduced, a more marked decrease is seen in late‐acting components including *DFR*, *LAR*, *ANR*, *MATE*, and *AHA10*. This observation aligns with earlier research suggesting that the loss of anthocyanin pigmentation is often linked to *cis*‐ and/or *trans*‐regulatory changes in enzymatic genes (Shimada *et al*., 2004, 2005, 2007; Streisfeld & Rausher, 2011; Hatlestad *et al*., 2014; Larter *et al*., 2018; Sakuta *et al*., 2021; Wheeler *et al*., 2022, 2023). Interestingly, not all flavonoid biosynthesis genes follow this trend. For example, consistent expression levels of *FLS* and *FNS*, along with glycosylation enzymes *A3GT*, *A5GT*, and *F3GT*, are maintained in betalain‐pigmented lineages. This indicates that the observed expression patterns are not merely artifacts or biases in the data. The substrate versatility of glycosylation enzymes has been well‐documented, and some are known to modify betalains (Vogt *et al*., 1999; Vogt, 2002). Therefore, the stable expression of these genes between the two pigment types is not unexpected. Lastly, the sustained high expression of *FLS* and *FNS* in betalain‐pigmented species may reflect the ongoing presence of flavonols and flavonones. This suggests that the metabolic flux toward flavonols and flavonones may account for the maintained expression levels of these genes.

The observed overall decline in gene expression across the flavonoid biosynthesis pathway, including early‐acting components, raises intriguing questions, especially because betalain‐pigmented species still synthesize flavonols and flavones. One possible explanation for this widespread reduction in gene expression may relate to a metabolic shift within core Caryophyllales, specifically from phenylalanine‐derived to tyrosine‐derived pathways (Lopez‐Nieves *et al*., 2018). This shift is potentially facilitated by a gene duplication event that led to a novel isoform of arogenate dehydrogenase (ADHα). Unlike its ancestral form, ADHα lacks feedback sensitivity and promotes tyrosine over phenylalanine production in heterologous assays using *Nicotiana benthamiana* (Lopez‐Nieves *et al*., 2018). Such an isoform could constrain the availability of phenylalanine, contributing to the observed reduced gene expression in the flavonoid pathway. Alternatively, the diminished demand for naringenin chalcone, owing to the absence of anthocyanin as a final product, might be sufficient to account for the lower expression levels.

### Repeated gene loss and convergent degeneration within the anthocyanin MBW transcription factor complex in betalain‐pigmented lineages

The evolutionary trajectory of the MYB‐bHLH‐WDR (MBW) complex within Caryophyllales presents intriguing features that merit close examination. Hatlestad *et al*. (2014) showed that BvPAP1 (here termed PAP1α) in *B. vulgaris* has lost the ability to interact with heterologous bHLH partners and attributed the loss of anthocyanins in *B. vulgaris* to this lost MYB‐bHLH interactivity. However, because Hatlestad *et al*. (2014) used heterologous *Arabidopsis* bHLH partners to test the BvPAP1/bHLH interaction, the fate of the native bHLH partners was never clearly articulated. Yet, the bHLH lineages, GL3 and MYC1, show interesting patterns of absence and/or loss. For example, we show that the GL3 ortholog, which normally partners with PAP1 to regulate anthocyanin production has been completely lost in *B. vulgaris*, reinforcing the concept that the canonical anthocyanin MBW complex has thoroughly disintegrated in all taxa within betalain transition 2 (Amaranthaceae). However, this is not the case for taxa associated with other transitions, for example, despite patchy recovery overall, GL3 homologs appear to be present in all data‐rich families and genomes within betalain transitions 3 and 4. The MYC1 lineage meanwhile shows an intriguing pattern, being consistently absent from all transcriptome and genome assemblies associated with betalain transition 3. Lastly, for comparison, the homologs of TT8 bHLH which partner with the MYB TT2 to regulate proanthocyanidin production are highly recovered in our data, consistent with the continued presence of proanthocyanidins in betalain‐pigmented lineages.

Hatlestad *et al*. (2014) showed that BvPAP1 alone can regulate betalain synthesis, independent of an MBW complex. However, here we report that orthologs of BvPAP1, that is the PAP1α lineage, are undetectable in any other betalain lineages and have been lost in genomes representing betalain transitions 3 and 4. In the absence of *BvPAP1* orthologs (the PAP1α lineage), one can reasonably infer that nonhomologous mechanisms regulating betalain pigmentation must exist in at least the whole‐genome betalain species (*M. crystallinum* and *C. gigantea*), and this inference may reasonably extend to all related taxa occurring within the same transitions to betalain pigmentation (i.e. transitions 3 and 4). Given the presence of PAP1α orthologs in both anthocyanic Kewaceae and Macarthuriaeae, one can infer at least two independent losses of PAP1α, which, in line with *TT19* losses, is consistent with multiple separate losses of anthocyanins. However, we show that there are two paralogous lineages of PAP1 in Caryophyllales. Although the PAP1α lineage appears to have been repeatedly lost, the reciprocal retention of the paralogous lineage PAP1β, in mirror image to the PAP1α losses, remains intriguing. Even more so, because PAP1β homologs show convergent patterns of residue substitutions, seen also in PAP1α at sites that have been shown to be influential in mediating MYB‐bHLH interactions (Sakuta *et al*., 2021), perhaps indicating a similar loss of PAP1/bHLH interaction in the PAP1β lineage. Although in contrast to lineages with PAP1α, canonical partners such as GL3 homologs are retained in taxa also possessing PAP1β. Although they have been implicated in anthocyanin regulation (Sakuta *et al*., 2021), the function and significance of PAP1β homologs is less well understood and merits further investigation.

### Conclusion

In summary, our bioinformatic analyses corroborate three of the four postulated genetic mechanisms responsible for the disappearance of anthocyanins in betalain‐pigmented lineages. Specifically, these mechanisms include the elimination of *TT19* orthologs (Hypothesis 1), diminished expression of flavonoid pathway elements such as *DFR* and *LDOX* (Hypothesis 3), and the degradation of the canonical MBW complex (Hypothesis 4). Notably, we demonstrate recurrent instances of *TT19* ortholog loss and MBW complex degeneration, often coinciding with shifts to betalain pigmentation. Some of these mechanisms seem more decisive than others in terms of anthocyanin loss, and the newly annotated role of lost *TT19* orthologs in anthocyanidin synthesis in particular overturn the long‐standing belief that all structural aspects of anthocyanin synthesis are maintained in betalain‐pigmented lineages. Although our data do not clarify the sequential order of these evolutionary changes, they significantly influence our understanding of the directionality in pigment evolution. Overall, our findings not only validate multiple evolutionary shifts from anthocyanin to betalain pigmentation but also question the likelihood of reversals from betalain to anthocyanin pigmentation. Broadly speaking, this research builds on the rich literature on the molecular evolution of the anthocyanin pathway, highlighting that late‐stage enzymatic steps and corresponding regulatory complexes emerge as prime targets for evolutionary modification at macroevolutionary timescales, consistent with trends observed at microevolutionary levels.

## Competing interests

None declared.

## Author contributions

BP and SFB conceived the work. WCY and JCC provided unpublished genomic resources. BP and NW‐H conducted analyses with support from JD, AC and YY. SFB, BP and NW‐H prepared the figures. SFB and NW‐H wrote the manuscript, with methods contributed by BP. All authors read and approved the manuscript. BP and NW‐H are joint first authors for this work.

## Supporting information


**Fig. S1** Illustration of the cross‐species gene expression calculation that forms the basis of Fig. 6.
**Fig. S2** Phylogenetic trees of the 18 flavonoid biosynthesis and flavonoid transport genes.
**Fig. S3** Summary of flavonoid biosynthesis gene duplications in the Caryophyllales.
**Fig. S4** Heatmap of frequency of presence of flavonoid biosynthesis genes in 357 Caryophyllales species summarized at the family level.
**Fig. S5** Plots of RELAX alternative model fits showing dN/dS and site proportions of each site class for reference anthocyanin branches and test betalain branches.
**Fig. S6** Phylogenetic trees of six components that can contribute to the MBW complex.
**Fig. S7** Analyses of *Mirabilis jalapa* ANS gene copies.
**Table S1** Comparison of RNA‐Seq tissue types between anthocyanin‐pigmented and betalain‐pigmented plants.
**Table S2** Values for the ratio of synonymous and nonsynonymous substitutions between 23 genes from anthocyanin‐pigmented and genes from betalain‐pigmented taxa.
**Table S3** Log likelihoods, information criteria, and parameter estimates of null, alternative, and partitioned descriptive models for RELAX analyses.
Please note: Wiley is not responsible for the content or functionality of any Supporting Information supplied by the authors. Any queries (other than missing material) should be directed to the *New Phytologist* Central Office.

## Data Availability

RNA‐Seq data sets analyzed in this study are available at the SRA/ENA. A list of the analyzed data sets, Fasta files containing bait sequences and sequences identified in this study, and Python scripts developed for this study are available at github: https://github.com/bpucker/CaryoAnthoBlock.
