# Supplementary material for: Multiple mechanisms explain loss of anthocyanins from betalain‐pigmented Caryophyllales, including repeated wholesale loss of a key anthocyanidin synthesis enzyme
Source: New Phytol. 2023 Oct 28;241(1):471–89. doi: 10.1111/nph.19341 (PMC10952170; doi:10.1111/nph.19341)
Supplement: Supplementary file 1 — Fig. S1 Illustration of the cross‐species gene expression calculation that forms the basis of Fig. 6. Fig. S2 Phylogenetic trees of the 18 flavonoid biosynthesis and flavonoid transport genes. Fig. S3 Summary of flavonoid biosynthesis gene duplications in the Caryophyllales. Fig. S4 Heatmap of frequency of presence of flavonoid biosynthesis genes in 357 Caryophyllales species summarized at the family level. Fig. S5 Plots of RELAX alternative model fits showing dN/dS and site proportions of each site class for reference anthocyanin branches and test betalain branches. Fig. S6 Phylogenetic trees of six components that can contribute to the MBW complex. Fig. S7 Analyses of Mirabilis jalapa ANS gene copies. Table S1 Comparison of RNA‐Seq tissue types between anthocyanin‐pigmented and betalain‐pigmented plants. Table S2 Values for the ratio of synonymous and nonsynonymous substitutions between 23 genes from anthocyanin‐pigmented and genes from betalain‐pigmented taxa. Table S3 Log likelihoods, information criteria, and parameter estimates of null, alternative, and partitioned descriptive models for RELAX analyses. Please note: Wiley is not responsible for the content or functionality of any Supporting Information supplied by the authors. Any queries (other than missing material) should be directed to the New Phytologist Central Office. [file NPH-241-471-s001.pdf]

***New Phytologist* Supporting Information**

Article title: **Evolutionary blocks to anthocyanin accumulation and the loss of an anthocyanin carrier protein in betalain-pigmented Caryophyllales**

Authors: **Boas Pucker, Nathanael Walker-Hale, Won C. Yim, John Cushman, Alexandra Crum, Ya Yang, Samuel Brockington**

Article acceptance date: 23rd September 2023

The following Supporting Information is available for this article:

Figure S1. Illustration of the cross-species gene expression calculation that forms the basis of Fig. 6.

Figure S2. Phylogenetic trees of the 18 flavonoid biosynthesis and flavonoid transport genes.

Figure S3. Summary of flavonoid biosynthesis gene duplications in the Caryophyllales

Figure S4. Heatmap of frequency of presence of flavonoid biosynthesis genes in 359 Caryophyllales species summarized at the family level.

Figure S5. Plots of RELAX alternative model fits showing dN/dS and site proportions of each site class for reference anthocyanin branches and test betalain branches.

Figure S6. Phylogenetic trees of 6 components that can contribute to the MBW complex.

Figure S7. Analyses of *Mirabilis jalapa* ANS gene copies.

Table S1. Comparison of RNA-seq tissue types between anthocyanin-pigmented and betalain-pigmented plants.

Table S2. Table showing values for ratio of synonymous and non-synonymous substitutions between 17 genes from anthocyanin-pigmented and genes from betalain-pigmented taxa, (excluding AN9 as no recovered from betalain lineages) averaged over all codons in the alignment.

Table S3. Table showing log likelihoods, information criteria and parameter estimates of RELAX null, alternative and partitioned descriptive models between 23 genes from anthocyanin-pigmented and genes from betalain-pigmented taxa, (excluding AN9 as no recovered from betalain lineages).

Supplementary Figure S1. Illustration of the cross-species gene expression calculation that forms the basis of Fig. 6. These illustrations explains how the values of one gene (geneX) are calculated. All isoforms of one gene are identified across all the analyzed species. For each sample, the abundances of all isoforms of a gene are added up (4+4=8, for sample 1 of species1). The resulting values of all samples are used to calculate an average value per species (8+4+8+6=26, 26/4 = 6.5, for species 1). The values of all anthocyanin-pigmented species are displayed in the blue violin plot and the values of all betalain-pigmented species are displayed in the magenta violin plot.

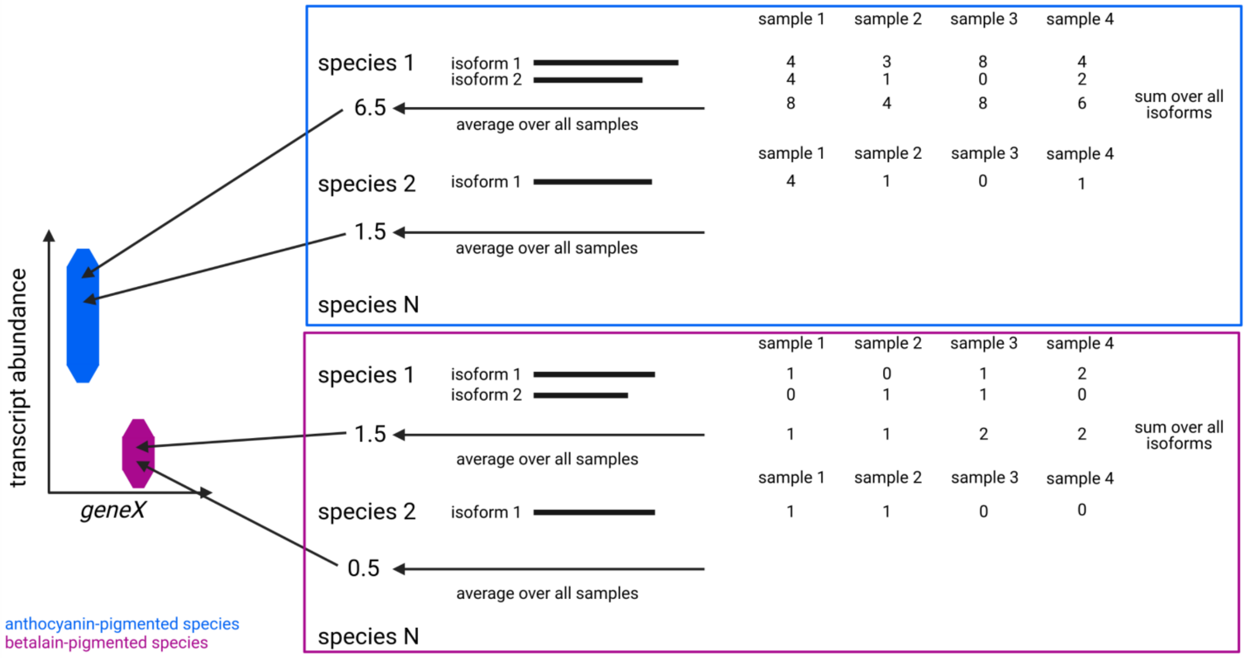

Supplementary Figure S2. Phylogenetic trees of 18 genes in the flavonoid biosynthesis and flavonoid transport steps were constructed by aligning peptide sequence with MAFFT, replacing amino acids with the corresponding codons, trimming to a minimal alignment column occupancy of 10%, inference of phylogeny with RAxML, and rooting by non-Caryophyllales outgroups. Blue = anthocyanin-pigmented lineages, pink = betalain-pigmented lineages. Numbers = support values for internal nodes, scale bars indicate branch lengths.

CHS

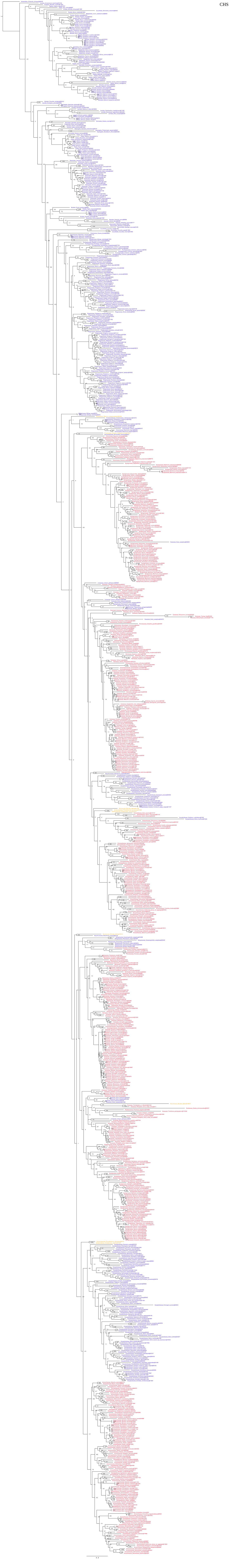

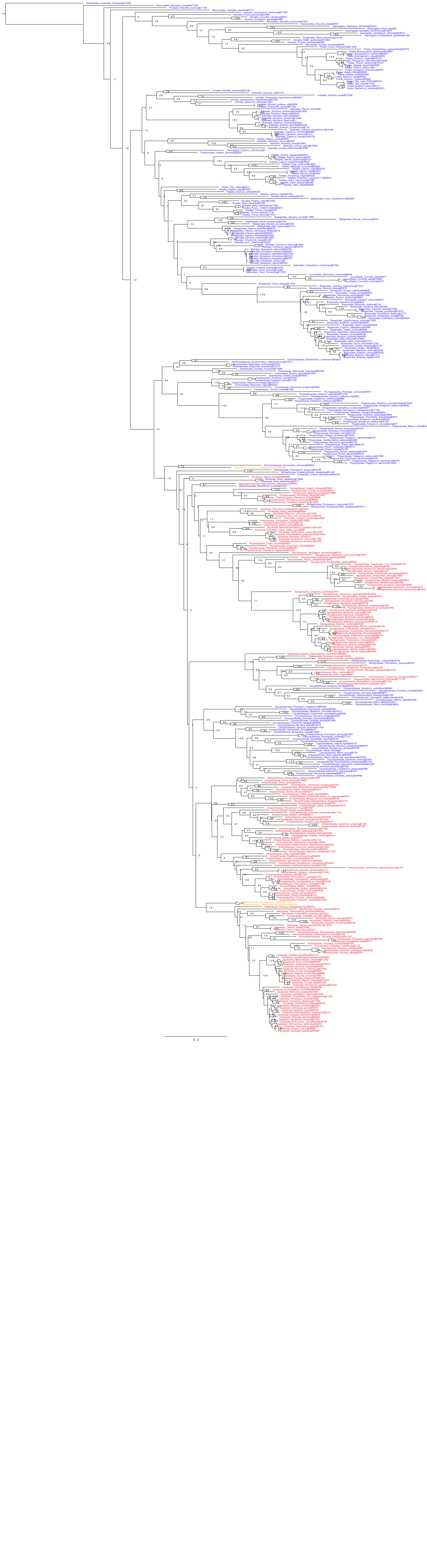

CHI

F3H

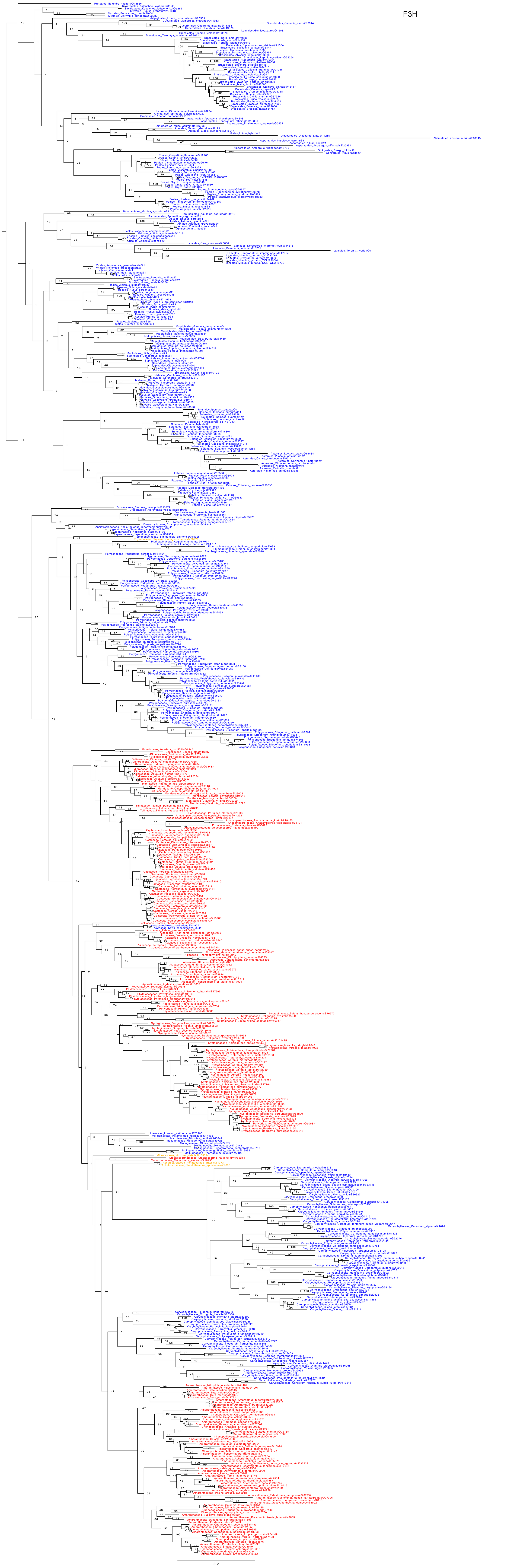

F3'H

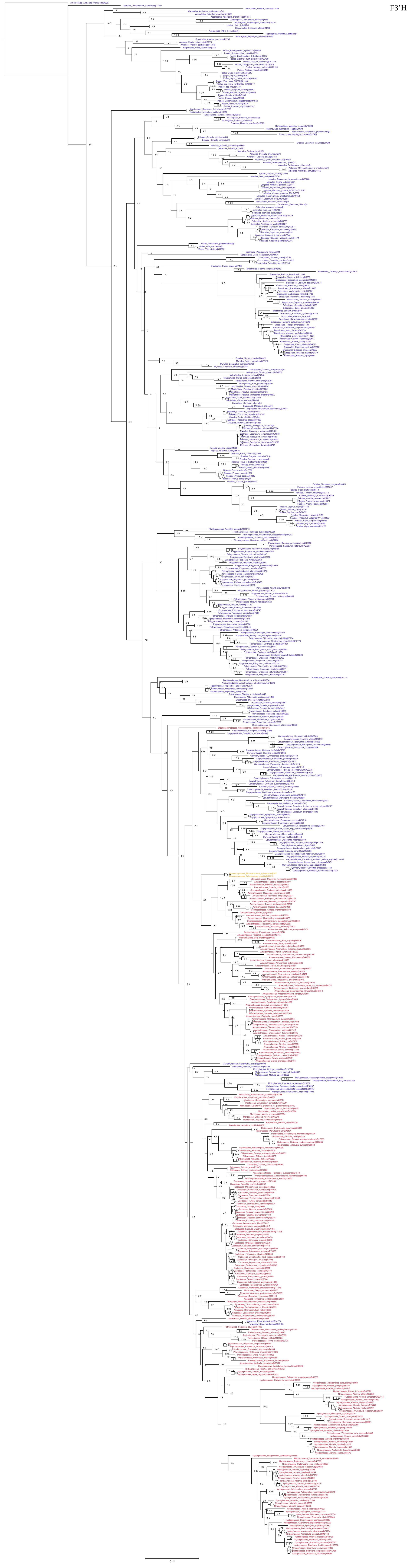

F3'5'H

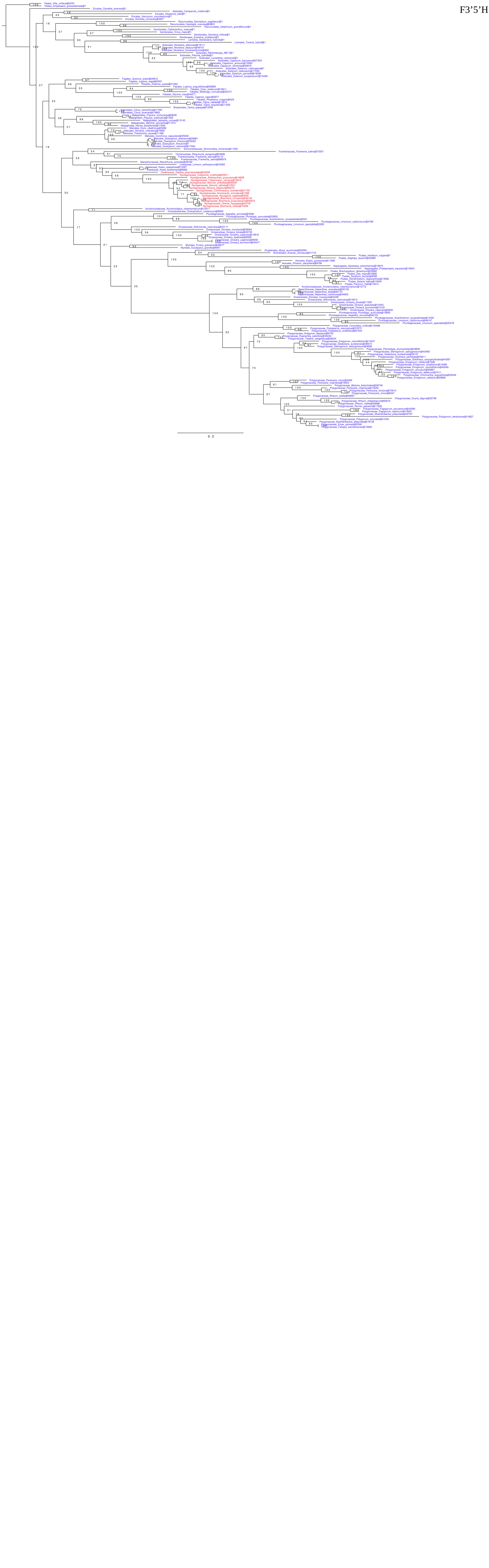

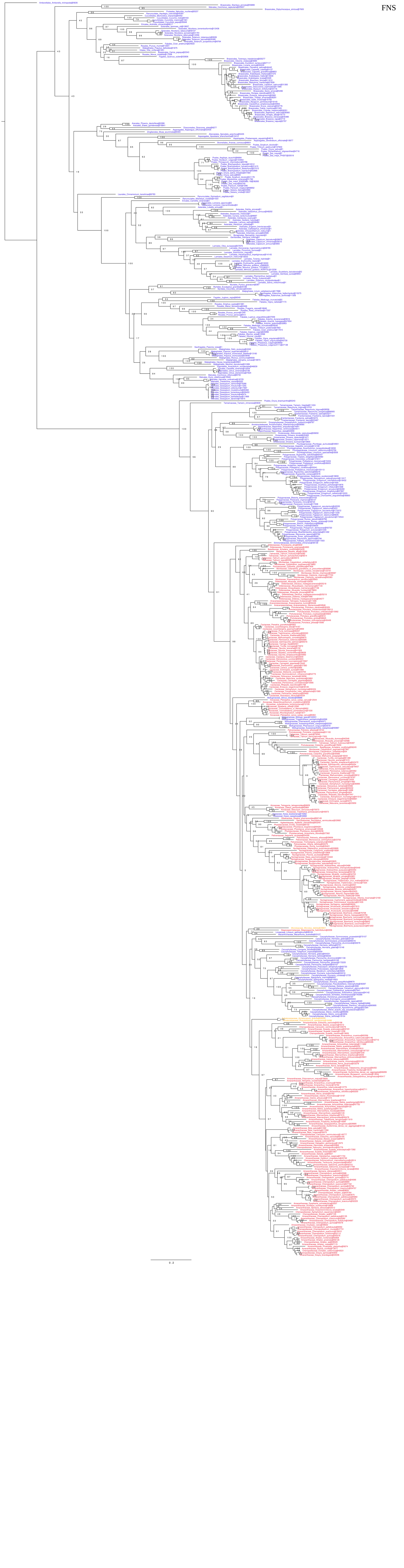
FNS

FLS

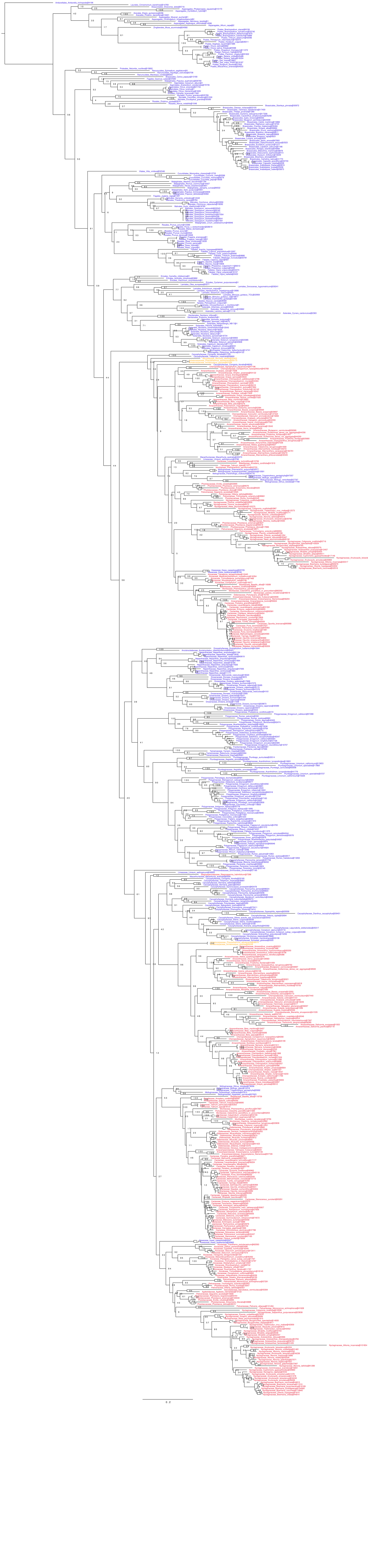

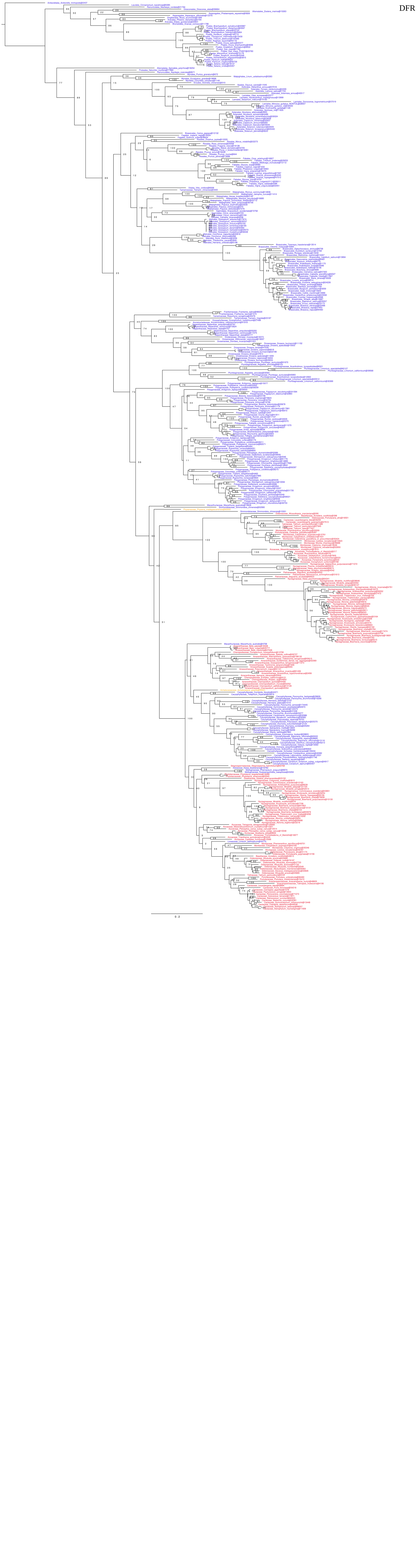
DFR

LDOX

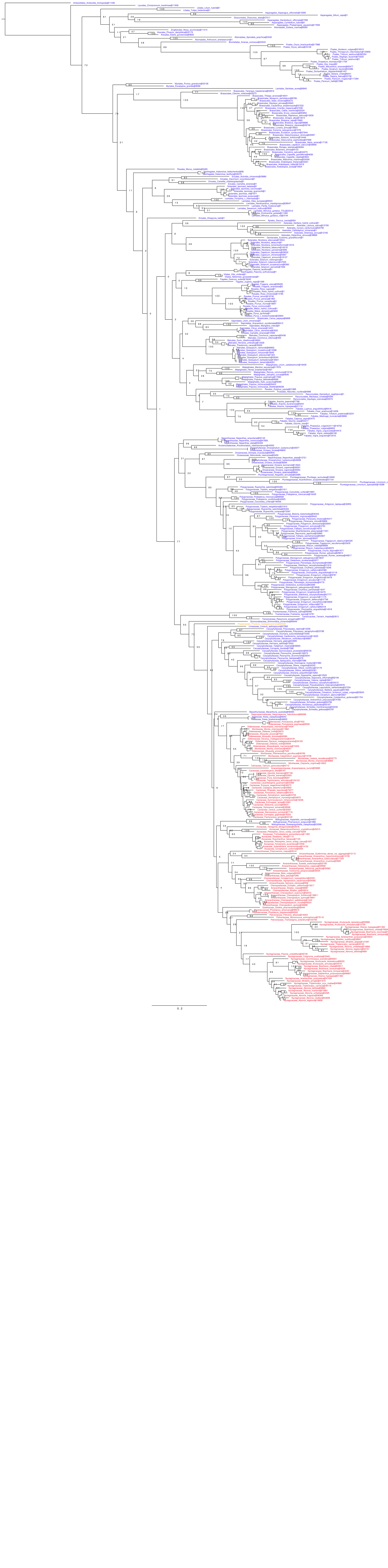

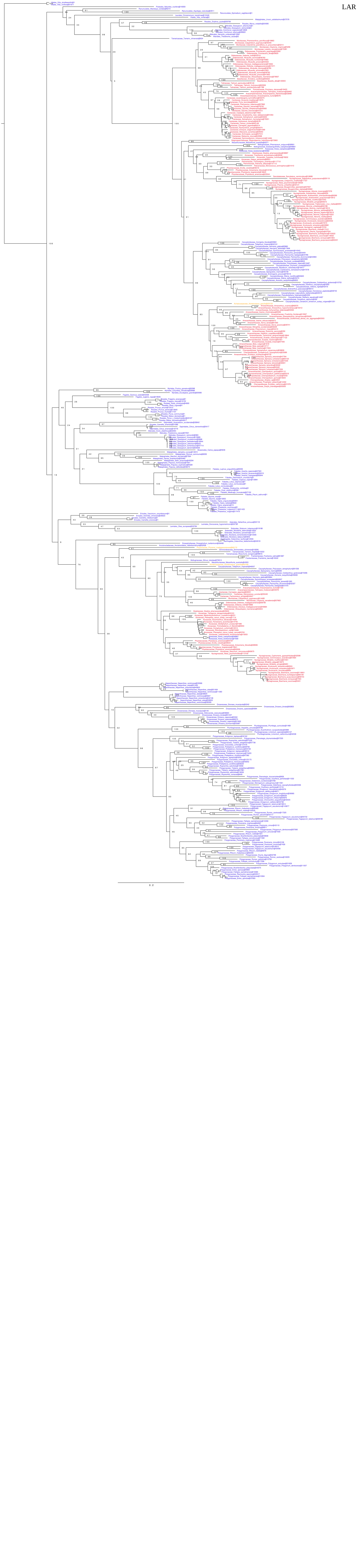
LAR

ANR

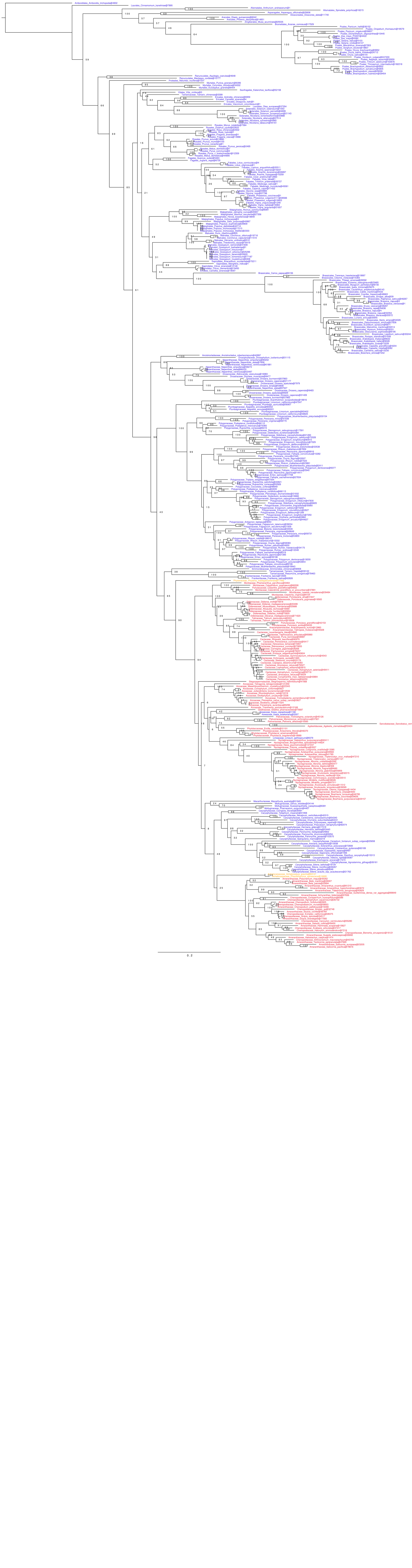

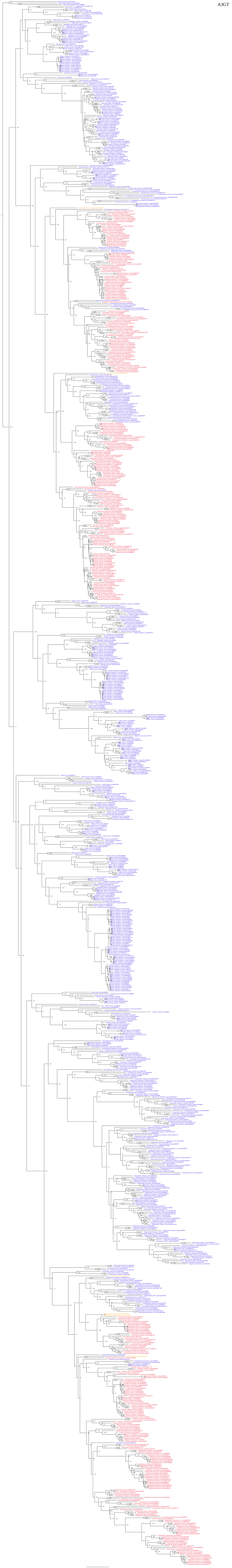
A3GT

A5GT

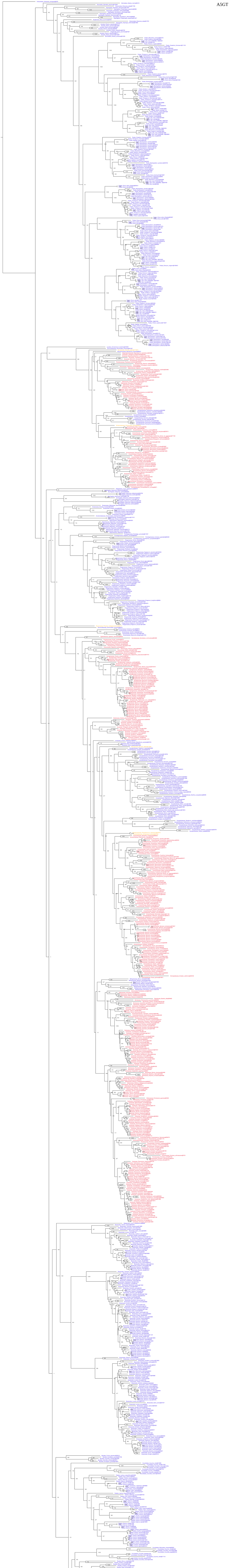

F3GT

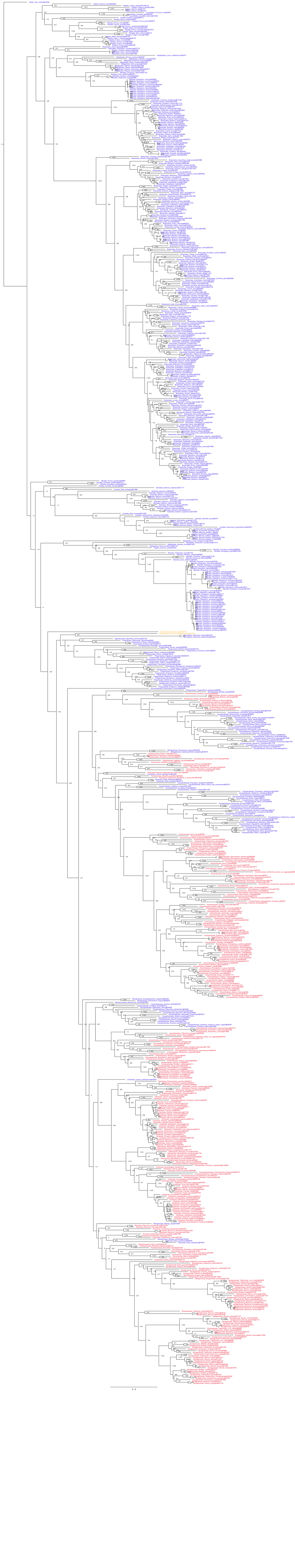

AN9/TT19

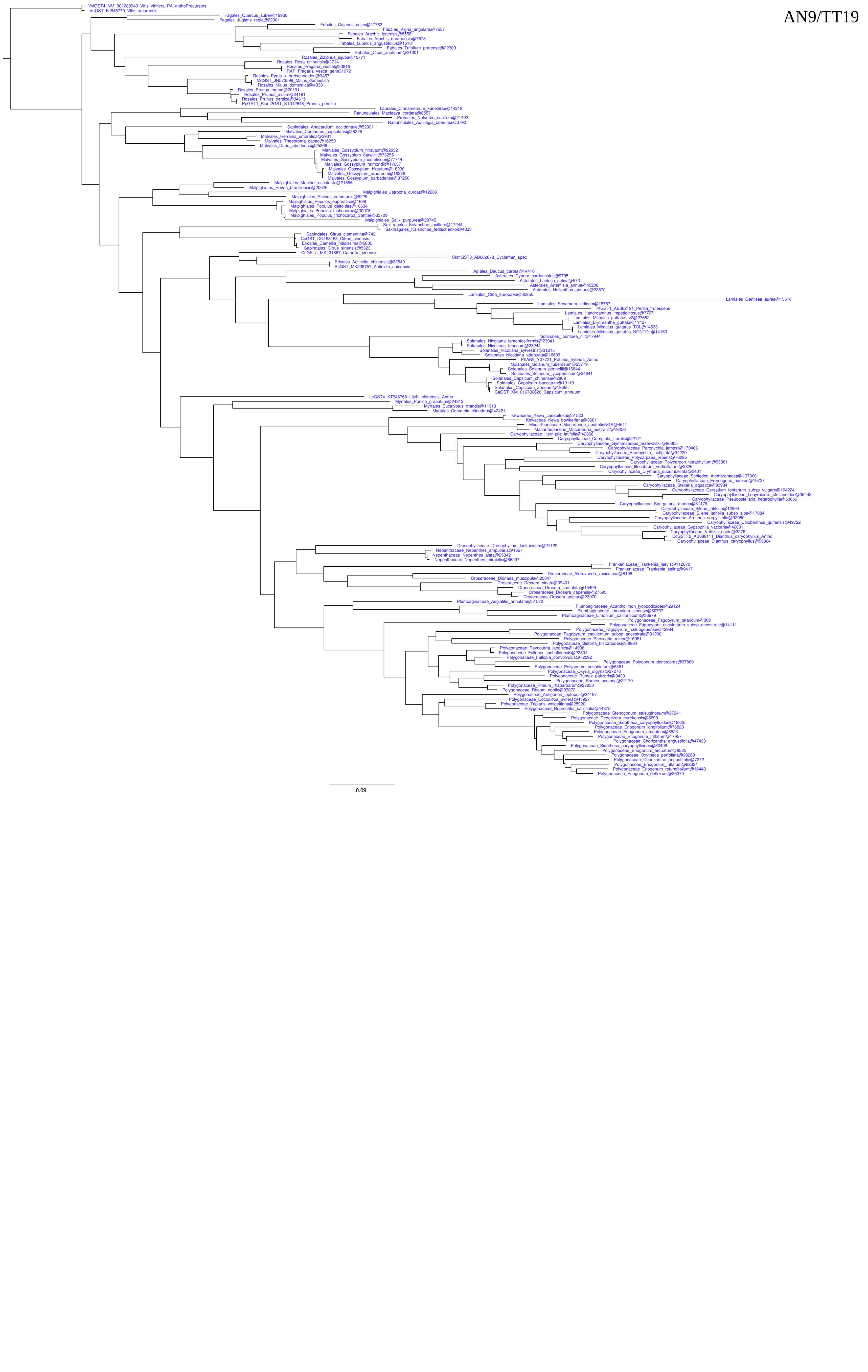

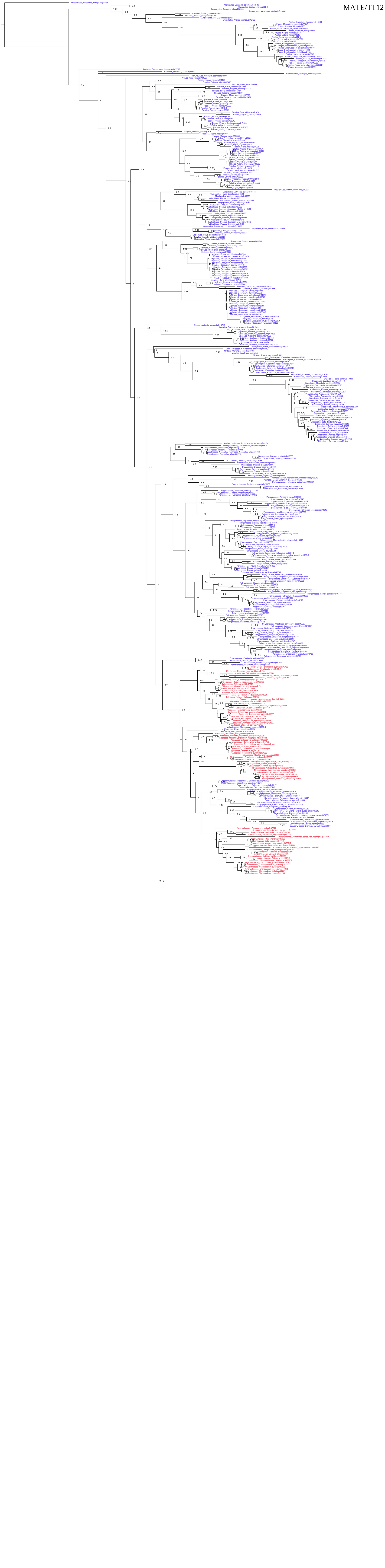
MATE/TT12

ABCC

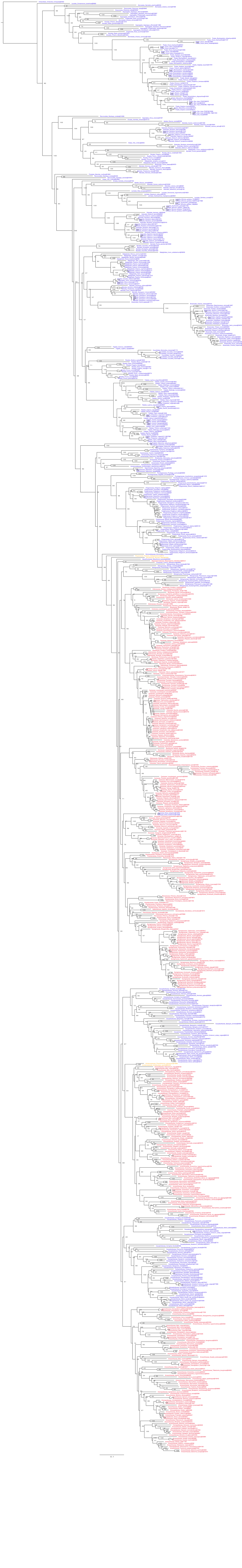

AHA10

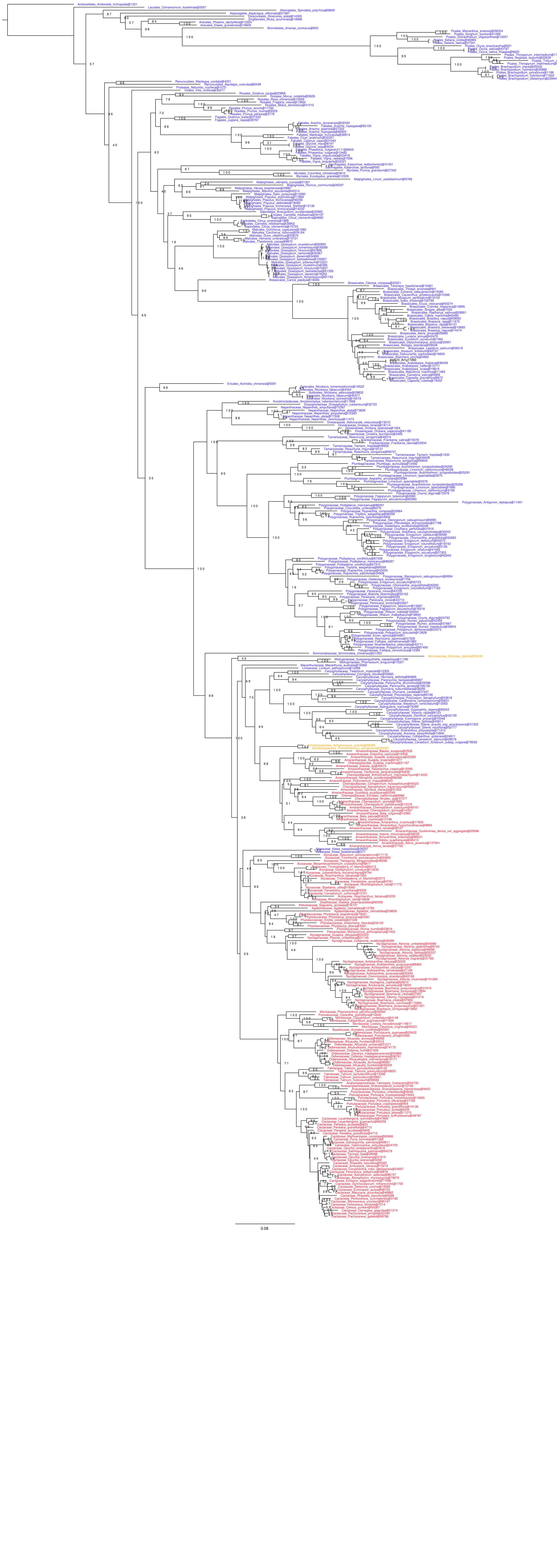

Supplementary Figure 3. Gene duplication events for flavonoid biosynthesis genes are mapped to a family-level phylogeny based on Walker *et al.*, 2018 and Sheehan *et al.,* 2020. The representation is restricted to deeper level gene duplications and excluded events below the genus level. CHS (naringenin-chalcone synthase), CHI (chalcone isomerase), FNS (flavone synthase), FLS (flavonol synthase), F3H (flavanone 3-hydroxylase), F3′H (flavonoid 3′-hydroxylase), F3′5′H (flavonoid 3′5′-hydroxylase), DFR (dihydroflavonol 4-reductase), LDOX (leucoanthocyanidin dioxygenase), TT19 (glutathione S-transferase), LAR (leucoanthocyanidin reductase), and ANR (anthocyanidin reductase), 3GT (anthocyanidin 3-O-gucosyltranferase), 5GT (anthocyanidin 5-O-glucosyltranferase), FGT (flavonoid 3-O-glycosyltransferase), MAT (proton antiporter), ABC (ATP binding cassette protein 1), and AHA (autoinhibited H(+)-ATPase isoform 10). Family names in blue = anthocyanin, pink = betalain, grey = unknown pigmentation status. Asterisks indicate families which are not represented or not well represented in the analyzed data set.

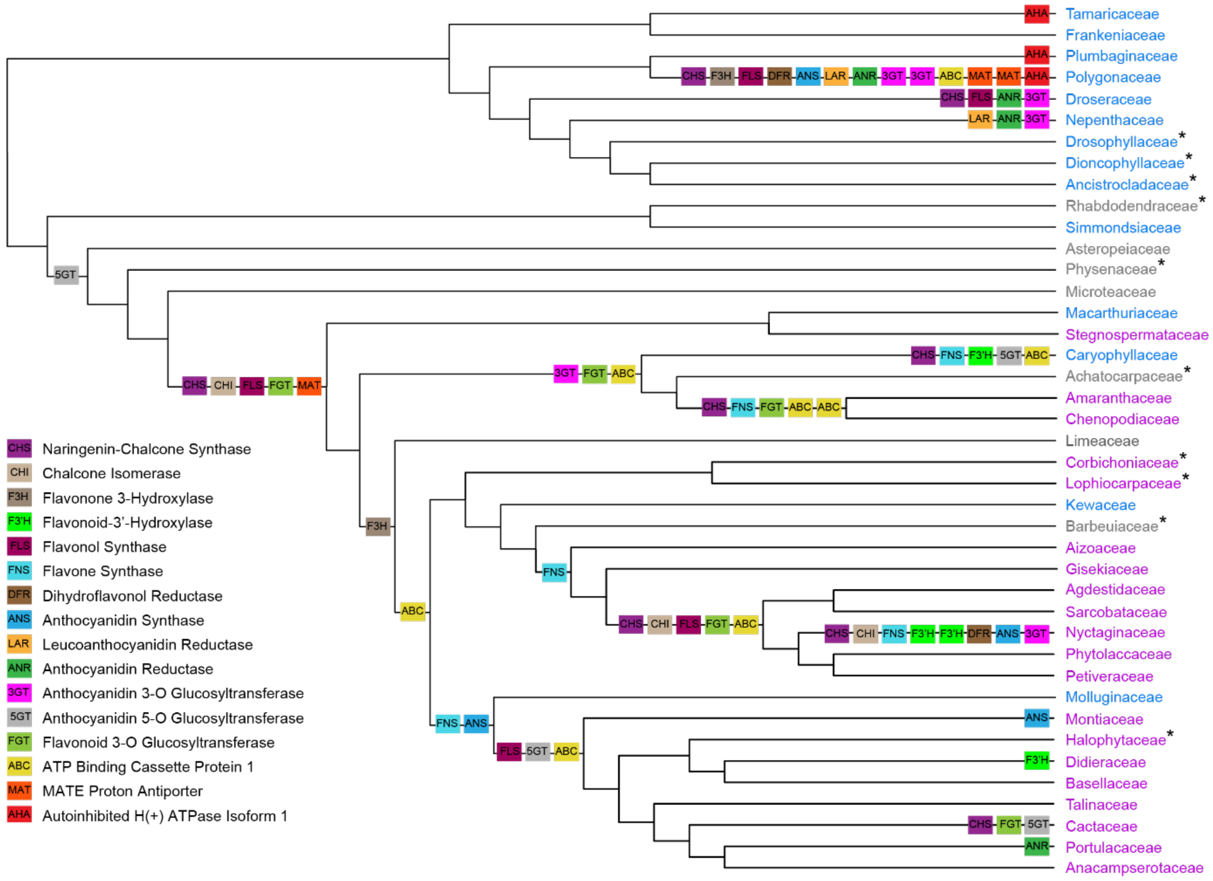

Supplementary Figure S4. Heatmap of frequency of presence of flavonoid biosynthesis genes in 357 Caryophyllales species summarized at the family level. Families are sorted by pigmentation state into anthocyanin- and betalain-pigmented (blue=anthocyanin, pink=betalain) to highlight the consistent differences between pigment types. Generally, most genes of the flavonoid biosynthesis are present in most families. Only *F3'5'H* and *TT19* are consistently missing from betalain-producing families. *CHS* (naringenin-chalcone synthase), *CHI* (chalcone isomerase), *FNS* (flavone synthase), *FLS* (flavonol synthase), *F3H* (flavanone 3-hydroxylase), *F3'H* (flavonoid 3′-hydroxylase), *F3'5'H* (flavonoid 3′5′-hydroxylase), *DFR* (dihydroflavonol 4-reductase), LDOX (leucoanthocyanidin dioxygenase), *TT19* (glutathione S-transferase) *LAR* (leucoanthocyanidin reductase), and *ANR* (anthocyanidin reductase), *GT* (glycosyltranferase), *MATE* (proton antiporter), *ABCC* (ATP binding cassette protein 1), and *AHA10* (Autoinhibited H(+)-ATPase isoform 10). Squares coloured by intensity to represent proportion of species in which gene is detected in 10% increments. Black = 100% of species have detectable genes through to white = not detected in transcriptome, white with a cross=absence confirmed in highly contiguous whole genome sequencing data. Numbers on the righthand side indicate number of transcriptome and genome assemblies sampled (*italics)* and number of species (bold).

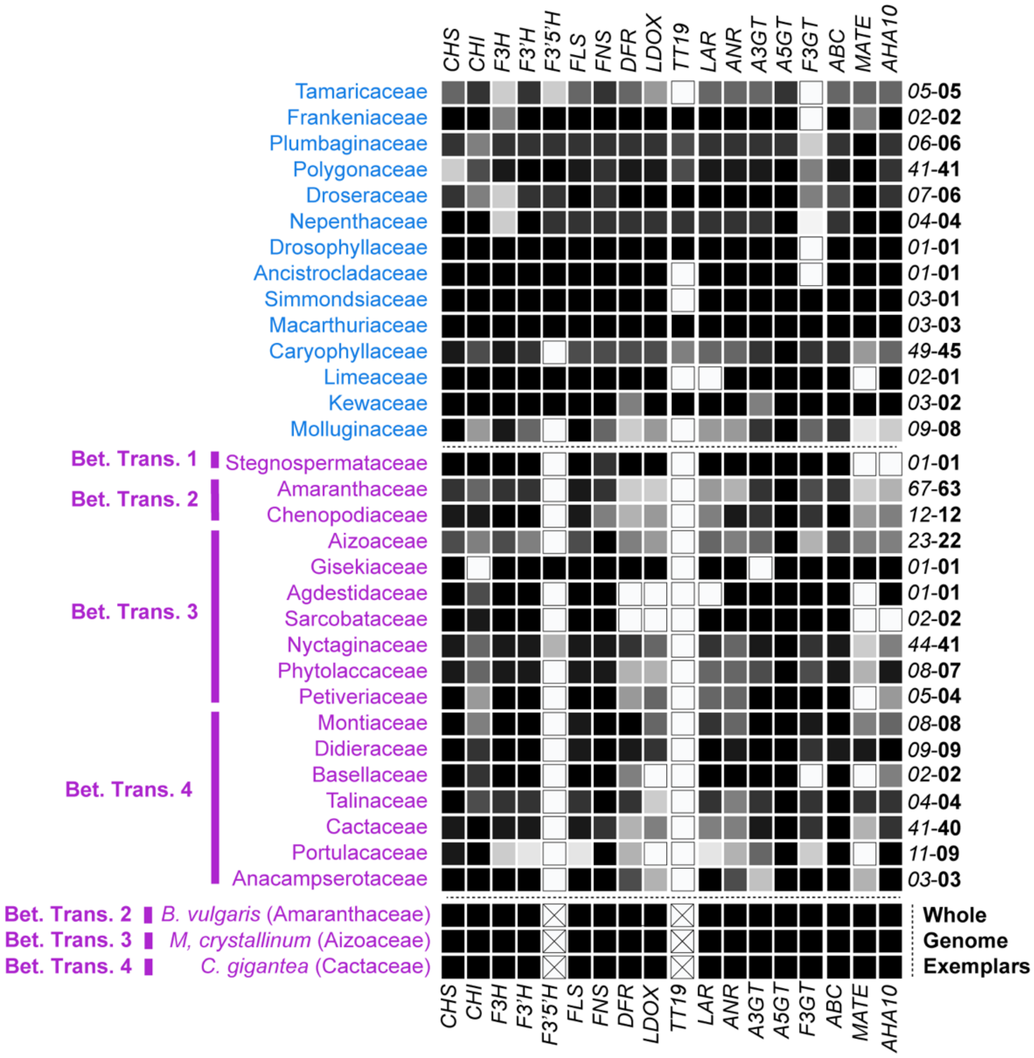

Supplementary Figure S5: plots of model fits from the RELAX alternative model. Each pair of bars represents the site proportion assigned to that site class for reference (blue) and test (pink) branch partitions, while the x-axis position gives the inferred dN/dS (omega) of that site partition. Note that site proportions are constrained to be equal between reference and test groups. In each case, K gives the inferred intensification/relaxation parameter, which corresponds to relaxed selection if $K < 1$ and intensified selection if $K > 1$. The P-value of the likelihood ratio test on one degree of freedom of the RELAX alternative against the RELAX null which constrains $K = 1$ is shown. AIC-c gives the small sampled-corrected Akaike Information Criterion for the RELAX alternative model.

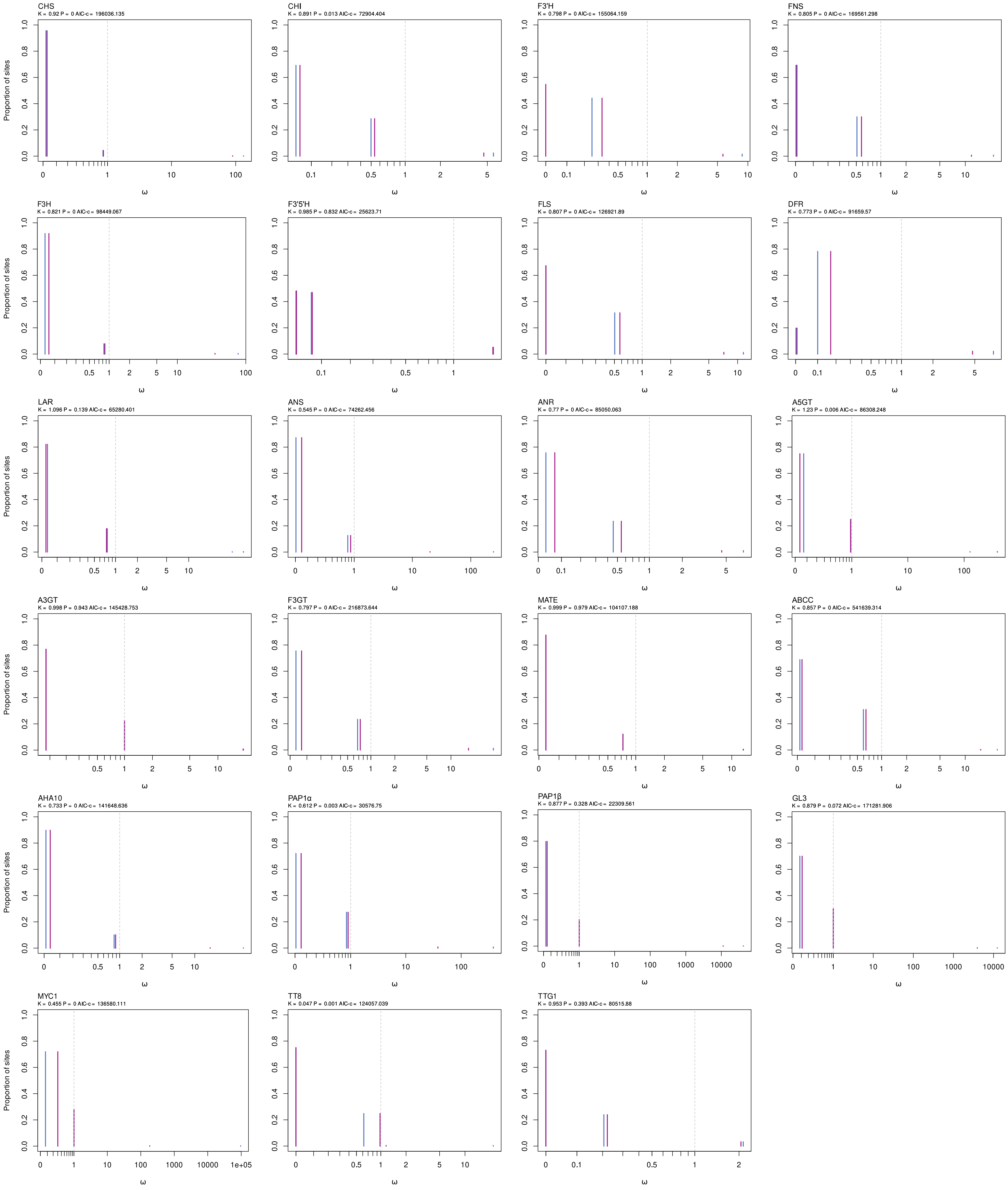
CHS
K = 0.92 P = 0 AIC-c = 196036.135
CHI
K = 0.891 P = 0.013 AIC-c = 72904.404
F3'H
K = 0.798 P = 0 AIC-c = 155064.159
FNS
K = 0.805 P = 0 AIC-c = 169561.298
F3H
K = 0.821 P = 0 AIC-c = 98449.067
F3'5'H
K = 0.985 P = 0.832 AIC-c = 25623.71
FLS
K = 0.807 P = 0 AIC-c = 126921.89
DFR
K = 0.773 P = 0 AIC-c = 91659.57
LAR
K = 1.096 P = 0.139 AIC-c = 65280.401
ANS
K = 0.545 P = 0 AIC-c = 74262.456
ANR
K = 0.77 P = 0 AIC-c = 85050.063
A5GT
K = 1.23 P = 0.006 AIC-c = 86308.248
A3GT
K = 0.998 P = 0.943 AIC-c = 145428.753
F3GT
K = 0.797 P = 0 AIC-c = 216873.644
MATE
K = 0.999 P = 0.979 AIC-c = 104107.188
ABCC
K = 0.857 P = 0 AIC-c = 541639.314
AHA10
K = 0.733 P = 0 AIC-c = 141648.636
PAP1α
K = 0.612 P = 0.003 AIC-c = 30576.75
PAP1β
K = 0.877 P = 0.328 AIC-c = 22309.561
GL3
K = 0.879 P = 0.072 AIC-c = 171281.906
MYC1
K = 0.455 P = 0 AIC-c = 136580.111
TT8
K = 0.047 P = 0.001 AIC-c = 124057.039
TTG1
K = 0.953 P = 0.393 AIC-c = 80515.88
Proportion of sites
ω

Supplementary Figure S6. Phylogenetic trees of 6 regulatory genes were constructed by aligning peptide sequence with MAFFT, replacing amino acids with the corresponding codons, trimming to a minimal alignment column occupancy of 10%, inference of phylogeny with RAxML, and rooting by non-Caryophyllales outgroups. Numbers represent support values for internal nodes. Scale bars represent branch lengths.

FIGURE S6
SUBGROUP6 (PAP1) + SaUBGROUP15 MYBS

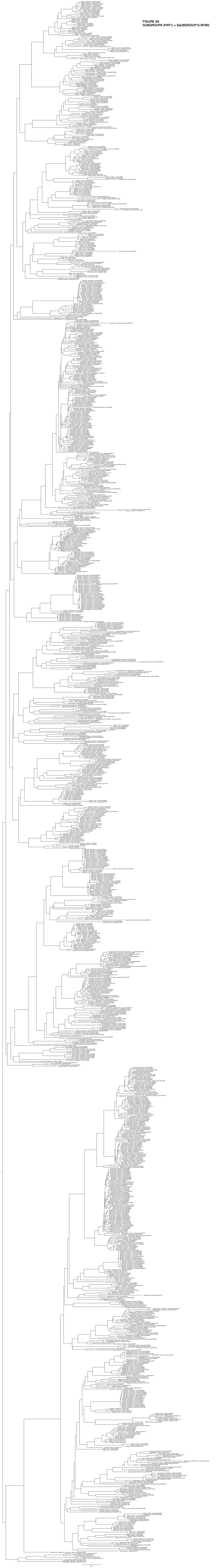

FIGURE S6
PAP1a

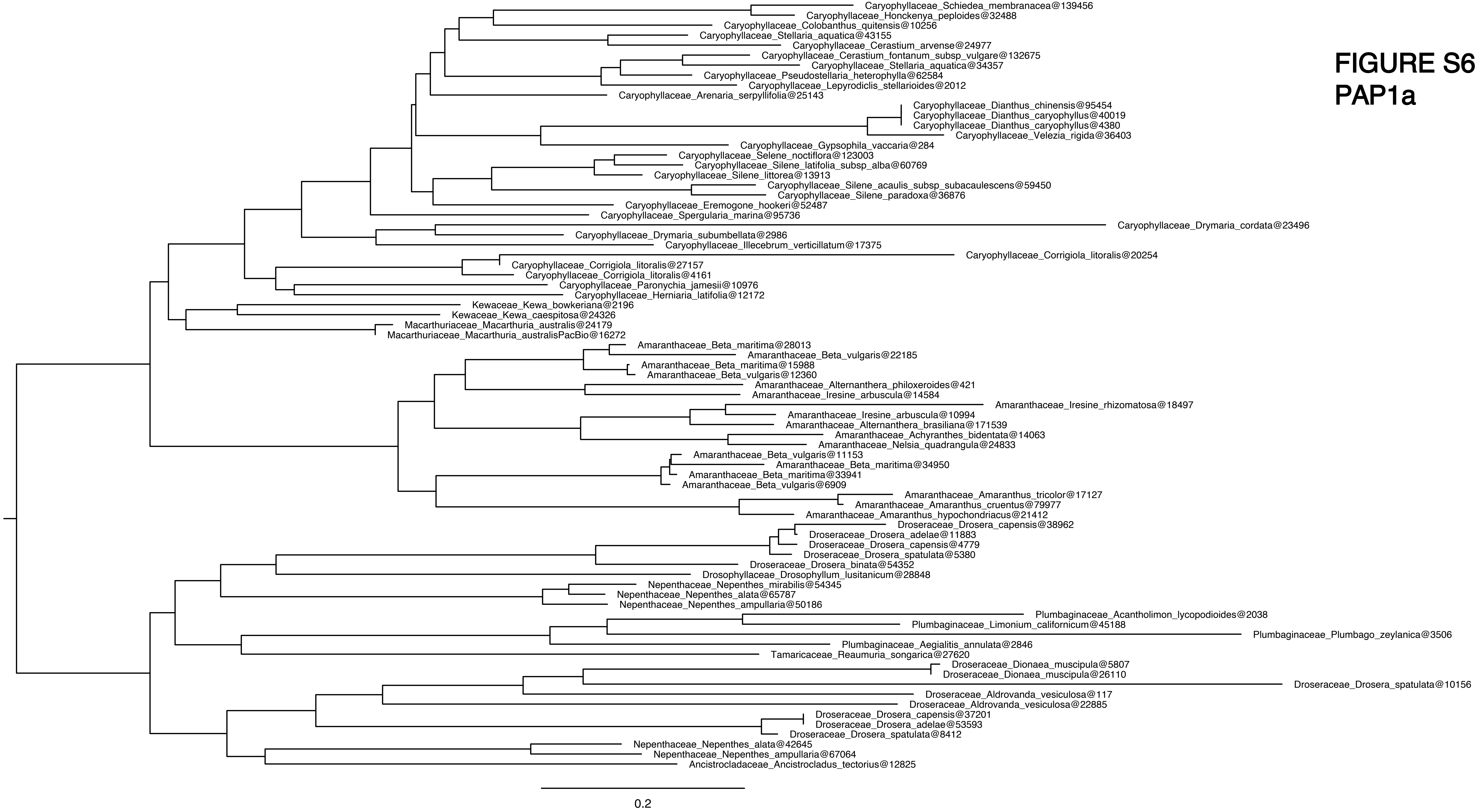

FIGURE S6
PAP1b

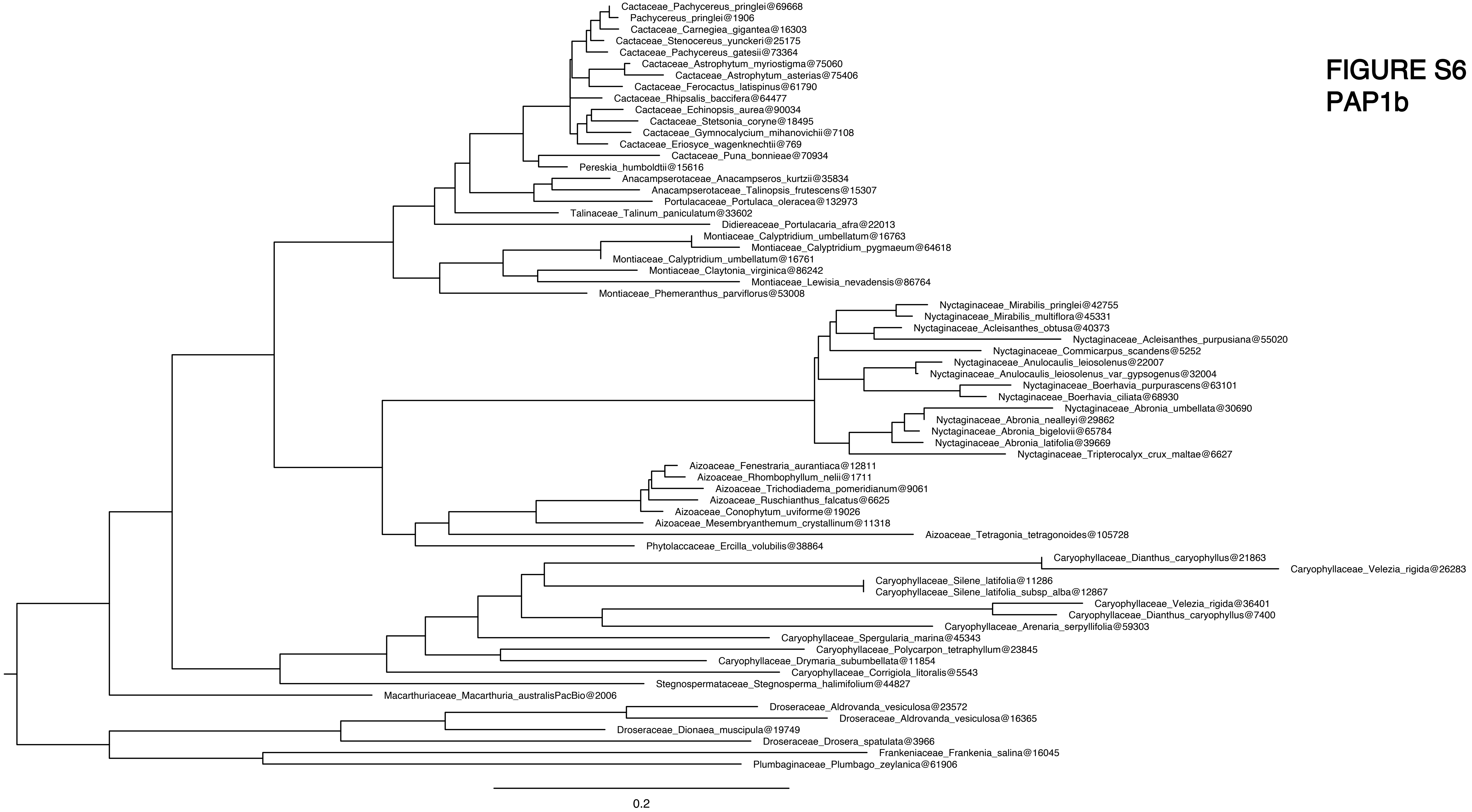

FIGURE S6
GL3

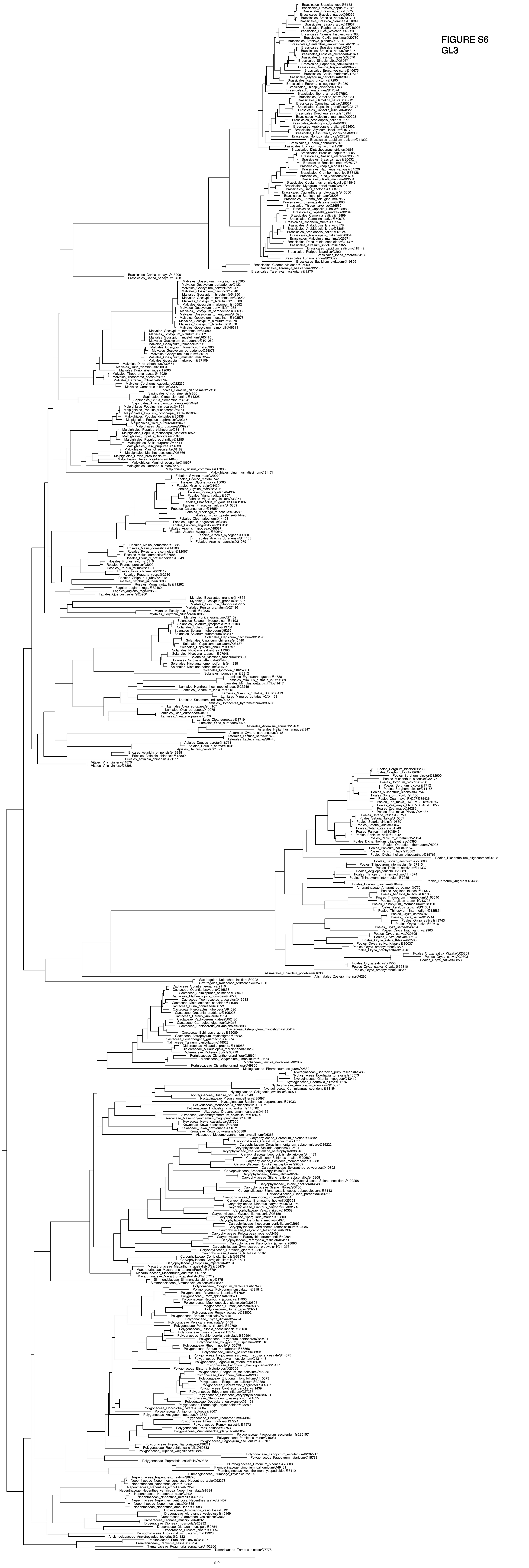

FIGURE S6
TTG1

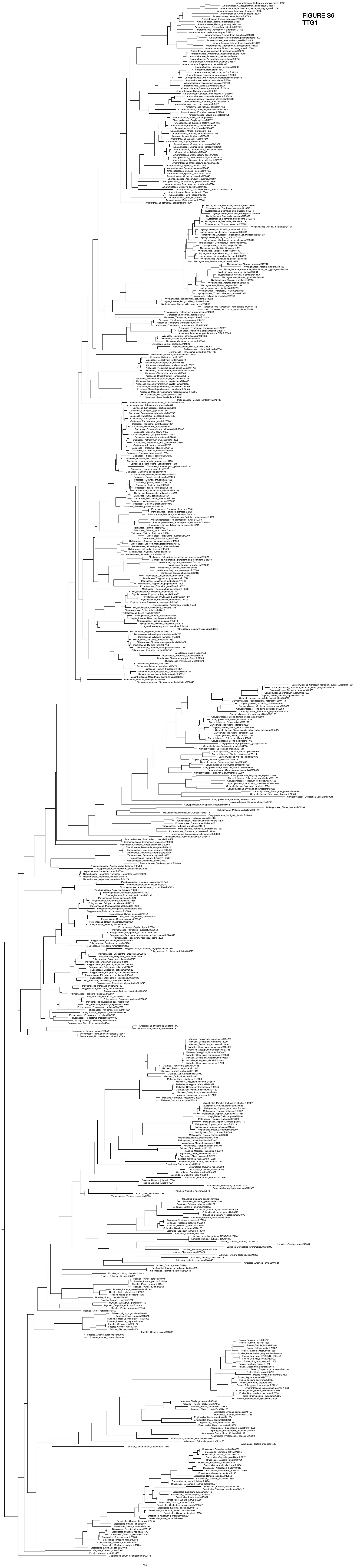

FIGURE S6
MYC1

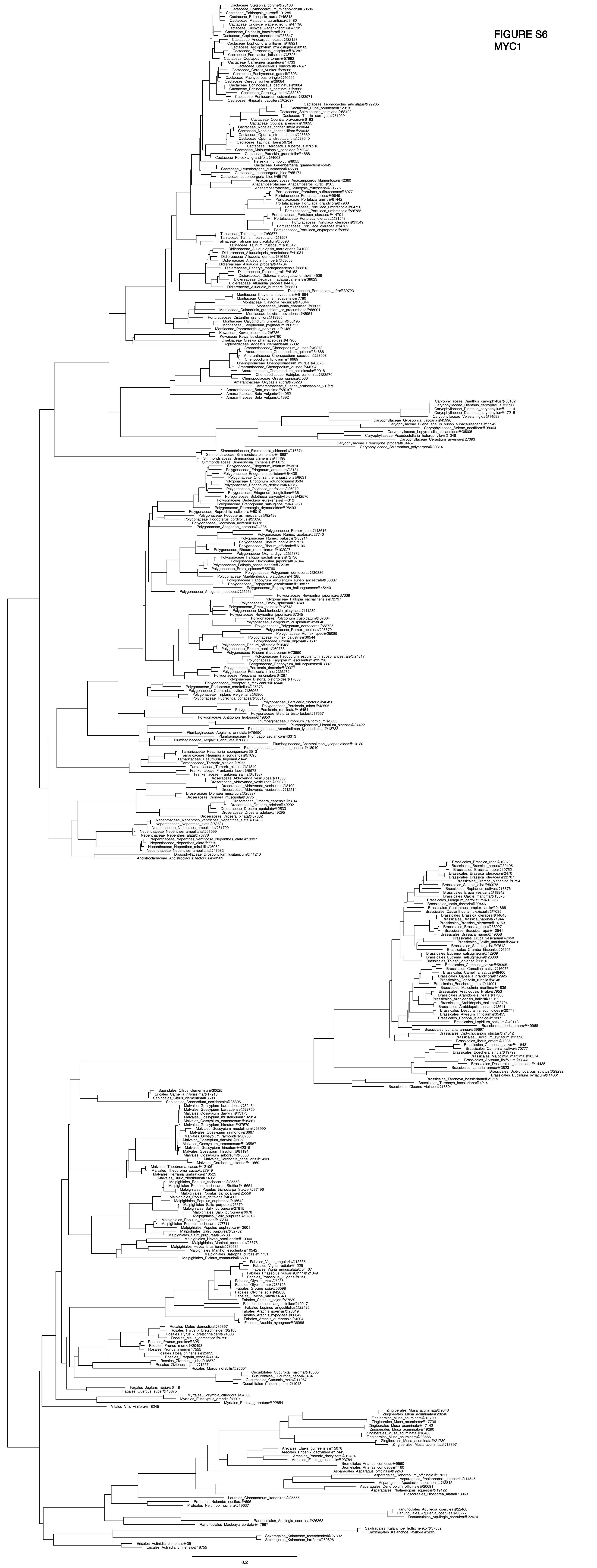

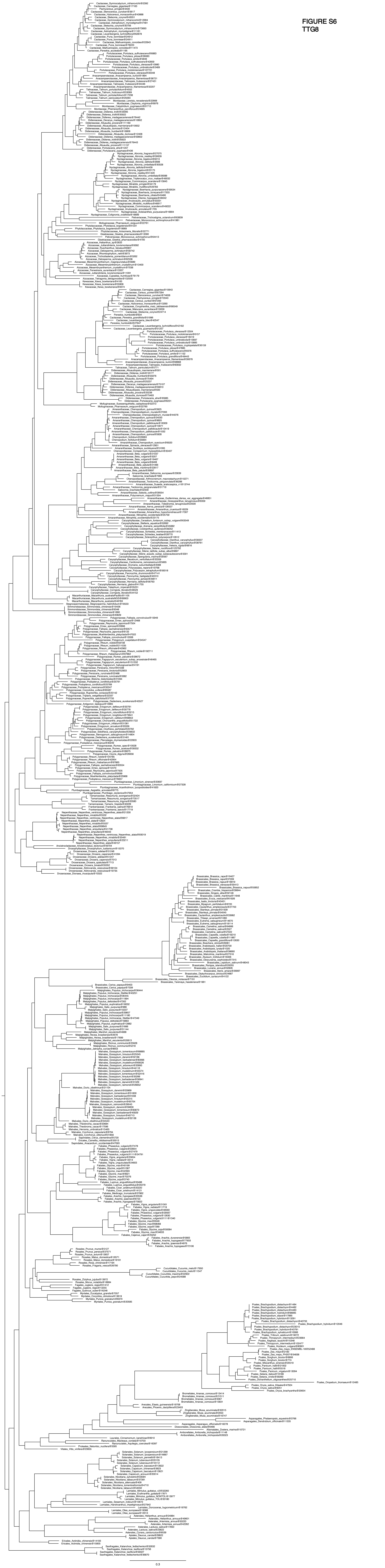

FIGURE S6
TTG8

Supplementary Figure S7. Comparison of truncated and full length ANS sequences in Mirabilis jalapa. (a) The phylogenetic positions of ANS(1) and ANS(2) indicates a gene duplication in the Nyctaginaceae. ANS sequences are highlighted in red. Two ANS clades are distinguished by color: blue and pink, respectively. (b) The full length and the truncated ANS differ by a large gap in an import ANS domain as previously described by Polturak et al., 2018.

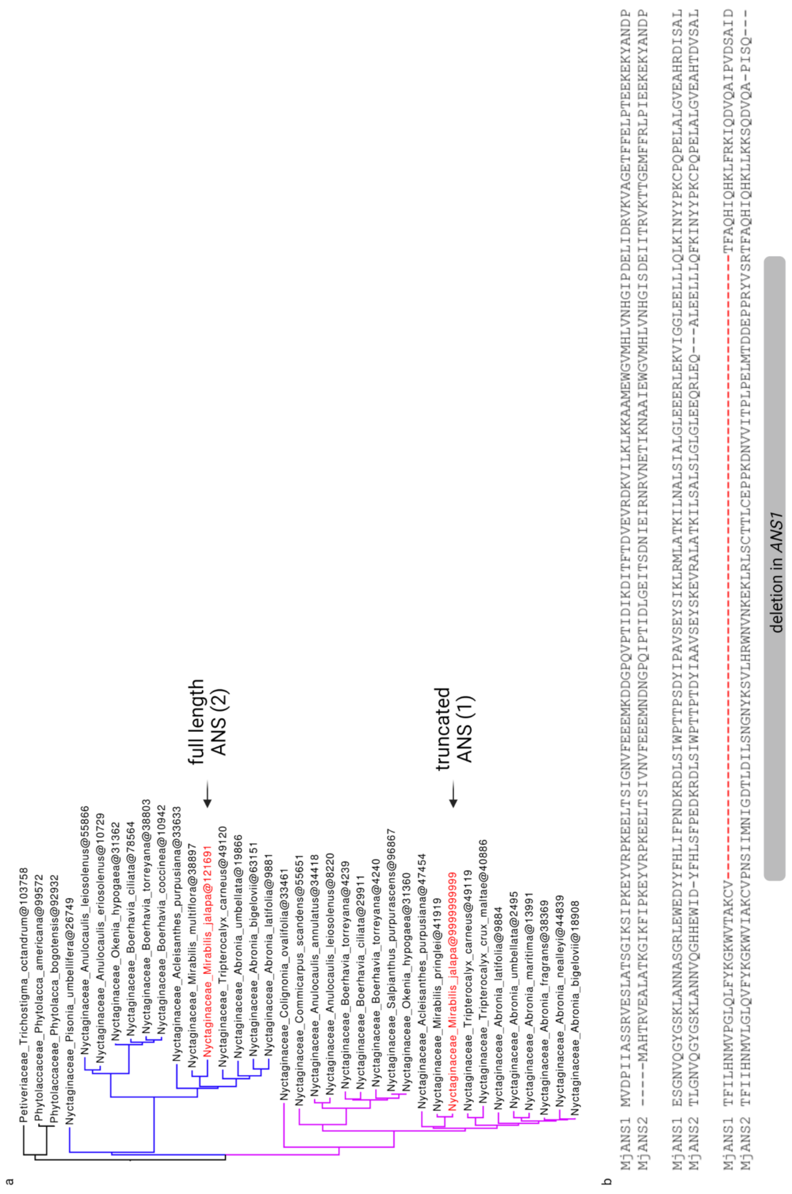

Table S1. Comparison of RNA-seq tissue types between anthocyanin-pigmented (anthocyanin) and betalain-pigmented (betalain) plants. The different groups used for the lineage specific comparison are all anthocyanin-pigmented species (A), betalain origin 2 (B2), betalain origin 3 (B3), and betalain origin 4 (B4). The metadata available for the analyzed samples were screened for frequently studied tissue types (leaf/leaves, flower, stem, root, seedling, seed).

Group	Leaf	Flower	Root	Seedling	Stem	Seed	unknown
anthocyanin	34	8	2	0	0	2	63
betalain	73	14	3	6	4	3	83
A	34	8	2	0	0	2	63
B2	25	1	1	4	1	3	25
B3	30	7	1	0	0	0	19
B4	18	6	1	2	3	0	39

Supplementary Table S2. Results of branch model analysis for 17 synthesis genes and 6 regulatory genes between anthocyanin-pigmented and betalain-pigmented lineages in Caryophyllales (excluding AN9). Model gives the two fitted models (null: M0, alternative branch), lnL gives the log-likelihood of the fitted model given the data and tree, ω_0 gives the overall ratio of non-synonymous to synonymous substitutions (dN/dS) across the tree for M0, and the dN/dS of background (anthocyanin) branches for the branch model. ω_1 gives the dN/dS of foreground (betalain) branches for the branch model. LRT P-value gives the P-value from the likelihood ratio test on chi-squared with one degree of freedom, bold lnL and P-values indicate significantly better fit for the branch model over M0 at $\alpha = 0.05$.

Gene	Model	lnL	ω_0	ω_1	LRT P-value
CHS	M0	-99524.67173	0.05496		
	branch	**-99518.07835**	0.05117	0.05811	**< 0.001**
CHI	M0	-36794.05138	0.22569		
	branch	**-36789.22219**	0.20752	0.24121	**< 0.01**
F3'H	M0	-78443.64699	0.15648		
	branch	**-78394.83032**	0.13338	0.1857	**< 0.001**
FNS2	M0	-85937.7626	0.16309		
	branch	**-85913.04465**	0.14339	0.17915	**< 0.001**
F3H	M0	-50091.61805	0.10029		
	branch	**-50068.98611**	0.08731	0.1171	**< 0.001**
F3'5'H	M0	-12911.20036	0.09956		
	branch	-12910.79834	0.09519	0.10443	0.3699
FLS	M0	-64473.56876	0.1745		
	branch	**-64446.54963**	0.15154	0.1982	**< 0.001**
DFR	M0	-46329.92424	0.1406		
	branch	**-46254.69801**	0.11121	0.19153	**< 0.001**
LAR	M0	-32914.584	0.15154		
	branch	-32914.43043	0.15469	0.14997	0.5794
ANS	M0	-37720.23281	0.10772		
	branch	**-37657.93976**	0.08479	0.14992	**< 0.001**
ANR	M0	-43073.44158	0.16649		
	branch	**-43038.3052**	0.14286	0.2107	**< 0.001**
A5GT	M0	-43522.57166	0.26503		
	branch	-43520.97329	0.28543	0.25939	0.0738
A3GT	M0	-73516.05429	0.27689		
	branch	-73491.69426	0.24061	0.30813	**< 0.001**
F3GT	M0	-110032.0929	0.2142		
	branch	**-109957.4936**	0.16661	0.24163	**< 0.001**
MATE	M0	-52679.34196	0.13272		
	branch	-52679.27106	0.13337	0.13098	0.7065
ABCCs	M0	-273426.6233	0.19551		
	branch	**-273388.3163**	0.17783	0.20768	**< 0.001**
AHA10	M0	-72008.30395	0.10541		
	branch	**-71968.30116**	0.09148	0.12941	**< 0.001**
PAP1α	M0	-15510.25826	0.1982		
	branch	**-15504.18668**	0.18721	0.26531	**< 0.001**
PAP1β	M0	-11258.04617	0.17534		
	branch	**-11254.25358**	0.14952	0.19344	**< 0.01**
GL3	M0	-86530.10502	0.31356		
	branch	-86528.71593	0.30995	0.33295	0.0956
MYC1	M0	-69122.13401	0.33513		
	branch	-69004.87415	0.27784	0.50614	**< 0.001**
TT8	M0	-62771.70247	0.16854		
	branch	**-62705.96561**	0.13745	0.21101	**< 0.001**

TTG1	M0	-40786.86565	0.08857		
	branch	-40786.66389	0.09005	0.08723	0.5253

Supplementary Table S3:

RELAX model fits. Model gives the null, alternative, or PD (partitioned descriptive) RELAX models. lnL is the log likelihood. AICc is the small sample-corrected Akaike Information Criterion. LRT P-val gives the P-value of the likelihood ratio test between the RELAX null and alternative models on 1 degree of freedom. K is the relaxation/intensification parameter. partition is the tree branch partition (reference = anthocyanin, test = betalain). ω_0, ω_1, ω_2 give the inferred dN/dS of each site class. p_0, p_1, p_2 give the site proportions of each site class. Note that the null model constrains K = 1, the alternative model constrains site proportions to be equal between reference and test branch partitions, but K is free to vary, and the partitioned descriptive model allows free estimation of ω and p in each partition.

Gene	Model	lnL	AICc	LRT P-val	K	Partition	ω_0	ω_1	ω_2	p_0	p_1	p_2
CHS	null	-97463.473	196054.723				0.025	0.851	116.131	0.955	0.043	0.002
	alternative	**-97453.168**	**196036.135**	**< 0.0001**	0.920	Reference	0.021	0.847	132.387	0.955	0.043	0.002
						Test	0.029	0.859	89.654	0.955	0.043	0.002
	PD	-97433.017	196003.916			Reference	0.022	1.000	147.076	0.960	0.037	0.003
						Test	0.030	0.744	122.892	0.954	0.045	0.001
CHI	null	-36199.998	72908.520				0.028	0.526	5.618	0.659	0.321	0.020
	alternative	**-36196.922**	**72904.404**	**0.013**	0.891	Reference	0.037	0.499	5.636	0.691	0.285	0.024
						Test	0.053	0.538	4.667	0.691	0.285	0.024
	PD	-36193.826	72906.358			Reference	0.065	0.488	4.320	0.768	0.195	0.037
						Test	0.000	0.540	8.608	0.586	0.404	0.010
F3'H	null	-77297.405	155112.871				0.000	0.282	5.764	0.549	0.435	0.016
	alternative	**-77272.041**	**155064.159**	**< 0.0001**	0.798	Reference	0.000	0.252	8.779	0.545	0.441	0.014
						Test	0.000	0.333	5.663	0.545	0.441	0.014
	PD	-77265.644	155059.430			Reference	0.009	0.281	8.555	0.607	0.380	0.013
						Test	0.000	0.507	10.993	0.660	0.333	0.007
FNS	null	-84510.117	169582.519				0.000	0.535	12.609	0.680	0.312	0.008
	alternative	**-84498.499**	**169561.298**	**< 0.0001**	0.805	Reference	0.002	0.510	21.472	0.693	0.300	0.007
						Test	0.007	0.582	11.816	0.693	0.300	0.007
	PD	-84498.037	169568.441			Reference	0.000	0.519	20.096	0.694	0.299	0.007
		-84498.037	169568.441			Test	0.004	0.543	10.745	0.673	0.318	0.009
F3H	null	-48963.282	98493.792				0.039	0.861	99.079	0.912	0.084	0.004
	alternative	**-48939.908**	**98449.067**	**< 0.0001**	0.821	Reference	0.031	0.834	77.195	0.918	0.078	0.004
						Test	0.058	0.861	35.500	0.918	0.078	0.004
	PD	-48935.411	98448.166			Reference	0.030	0.887	90.322	0.919	0.077	0.005
						Test	0.063	1.000	309.085	0.933	0.065	0.003
F3'5'H	null	-12748.535	25621.733				0.026	0.069	1.728	0.476	0.472	0.052
	alternative	-12748.513	25623.710	0.832	0.985	Reference	0.026	0.069	1.747	0.480	0.469	0.051

						Test	0.027	0.072	1.733	0.480	0.469	0.051
	PD	-12747.676	25630.125			Reference	0.027	0.070	2.001	0.495	0.454	0.051
						Test	0.040	0.071	1.649	0.516	0.439	0.045
FLS	null	-63133.887	126933.796				0.000	0.544	8.175	0.676	0.310	0.013
	alternative	**-63126.922**	**126921.890**	**< 0.001**	0.807	Reference	0.000	0.503	11.489	0.673	0.314	0.013
						Test	0.000	0.574	7.180	0.673	0.314	0.013
	PD	-63123.646	126923.436			Reference	0.000	0.573	12.197	0.704	0.285	0.010
						Test	0.000	0.468	7.472	0.612	0.374	0.014
DFR	null	-45613.501	91748.175				0.000	0.122	4.890	0.233	0.740	0.027
	alternative	**-45568.186**	**91659.570**	**< 0.0001**	0.773	Reference	0.001	0.100	7.513	0.198	0.781	0.021
						Test	0.006	0.168	4.758	0.198	0.781	0.021
	PD	-45559.165	91649.628			Reference	0.000	0.099	4.651	0.248	0.727	0.025
						Test	0.000	0.169	5.919	0.177	0.801	0.022
LAR	null	-32418.001	65280.565				0.032	0.753	50.730	0.824	0.174	0.002
	alternative	-32416.908	65280.401	0.139	1.096	Reference	0.036	0.766	39.215	0.821	0.178	0.002
						Test	0.026	0.747	55.698	0.821	0.178	0.002
	PD	-32414.611	65283.902			Reference	0.042	0.743	238.940	0.822	0.176	0.002
						Test	0.027	0.762	33.787	0.828	0.170	0.002
ANS	null	-36940.817	74356.452				0.000	0.751	36.106	0.832	0.165	0.002
	alternative	**-36892.807**	**74262.456**	**< 0.0001**	0.545	Reference	0.004	0.773	244.267	0.871	0.126	0.003
						Test	0.050	0.869	20.049	0.871	0.126	0.003
	PD	-36884.724	74254.387			Reference	0.000	0.688	28.403	0.857	0.140	0.003
						Test	0.037	1.000	1236361.2	0.862	0.138	0.001
ANR	null	-42310.132	85093.196				0.042	0.474	5.698	0.749	0.240	0.012
	alternative	**-42287.553**	**85050.063**	**< 0.0001**	0.770	Reference	0.032	0.455	7.152	0.756	0.234	0.010
						Test	0.070	0.545	4.549	0.756	0.234	0.010
	PD	-42278.591	85040.241			Reference	0.068	0.256	3.034	0.804	0.170	0.027
						Test	0.030	0.572	11.192	0.681	0.313	0.006
A5GT	null	-42962.013	86313.702				0.051	0.975	312.230	0.758	0.239	0.003

	alternative	**-42958.276**	**86308.248**	**0.006**	1.230	Reference	0.071	0.961	127.341	0.749	0.248	0.003
						Test	0.039	0.953	389.015	0.749	0.248	0.003
	PD	-42956.132	86312.029			Reference	0.058	0.998	141.866	0.735	0.260	0.004
						Test	0.046	0.965	431.830	0.762	0.236	0.003
A3GT	null	-72442.173	145426.740				0.079	1.000	18.771	0.768	0.224	0.008
	alternative	-72442.171	145428.753	0.943	0.998	Reference	0.079	1.000	18.686	0.768	0.224	0.008
						Test	0.079	1.000	18.553	0.768	0.224	0.008
	PD	-72435.213	145422.908			Reference	0.039	0.596	9.812	0.623	0.362	0.015
						Test	0.047	1.000	42.737	0.720	0.276	0.004
F3GT	null	-108166.819	216936.288				0.055	0.865	28.710	0.782	0.209	0.009
	alternative	**-108134.488**	**216873.644**	**< 0.0001**	0.797	Reference	0.034	0.676	33.676	0.754	0.233	0.013
						Test	0.067	0.732	16.518	0.754	0.233	0.013
	PD	-108121.689	216856.117			Reference	0.045	0.630	26.447	0.776	0.209	0.015
						Test	0.065	0.973	40.493	0.786	0.207	0.007
MATE	null	-51825.620	104105.172				0.034	0.731	13.205	0.875	0.120	0.005
	alternative	-51825.620	104107.188	0.979	0.999	Reference	0.034	0.731	13.200	0.875	0.120	0.005
						Test	0.034	0.731	13.166	0.875	0.120	0.005
	PD	-51817.428	104098.874			Reference	0.023	0.776	118.366	0.857	0.140	0.003
						Test	0.037	0.691	11.615	0.877	0.119	0.004
ABCC	null	-270473.836	541682.705				0.016	0.611	16.158	0.678	0.318	0.004
	alternative	**-270451.138**	**541639.314**	**< 0.0001**	0.857	Reference	0.014	0.595	24.078	0.688	0.308	0.004
						Test	0.026	0.641	15.272	0.688	0.308	0.004
	PD	-270445.694	541636.450			Reference	0.003	0.552	14.524	0.659	0.337	0.004
						Test	0.018	0.639	20.093	0.673	0.323	0.003
AHA10	null	-70612.600	141706.275				0.012	0.875	23.874	0.887	0.110	0.002
	alternative	**-70582.776**	**141648.636**	**< 0.0001**	0.733	Reference	0.012	0.839	44.147	0.897	0.101	0.002
						Test	0.038	0.880	16.042	0.897	0.101	0.002
	PD	-70578.112	141647.344			Reference	0.011	0.795	24.026	0.895	0.102	0.003
						Test	0.016	0.963	51.768	0.872	0.127	0.001

PAP1a	null	-15120.067	30583.840				0.000	0.854	291.173	0.704	0.289	0.007
	alternative	**-15115.500**	**30576.750**	**0.003**	0.612	Reference	0.008	0.840	380.784	0.720	0.272	0.008
						Test	0.052	0.898	38.032	0.720	0.272	0.008
	PD	-15114.958	30583.843			Reference	0.000	0.824	445.296	0.707	0.286	0.007
						Test	0.040	0.908	36.807	0.703	0.287	0.009
PAP1b	null	-11003.728	22308.479				0.032	1.000	44187.592	0.788	0.210	0.002
	alternative	-11003.249	22309.561	0.328	0.877	Reference	0.030	1.000	41425.817	0.797	0.201	0.002
						Test	0.047	1.000	11245.374	0.797	0.201	0.002
	PD	-11001.478	22314.184			Reference	0.000	0.669	9999999171.596	0.697	0.299	0.004
						Test	0.036	1.000	54663.500	0.780	0.218	0.002
GL3	null	-85286.406	171283.140				0.088	1.000	18948.123	0.703	0.295	0.002
	alternative	-85284.782	171281.906	0.072	0.879	Reference	0.081	1.000	12342.716	0.700	0.298	0.002
						Test	0.110	1.000	3931.153	0.700	0.298	0.002
	PD	-85279.305	171279.005			Reference	0.085	1.000	20211.395	0.704	0.294	0.002
						Test	0.077	1.000	19105.591	0.664	0.335	0.001
MYC1	null	-68137.129	136757.661				0.048	1.000	89106.487	0.642	0.356	0.002
	alternative	**-68047.348**	**136580.111**	**< 0.0001**	0.455	Reference	0.072	1.000	96254.023	0.719	0.279	0.002
						Test	0.302	1.000	185.264	0.719	0.279	0.002
	PD	-68037.637	136568.736			Reference	0.060	1.000	87556.959	0.711	0.287	0.002
						Test	0.050	1.000	85705.843	0.474	0.524	0.002
TT8	null	-61759.159	124066.038				0.011	0.628	17.488	0.733	0.262	0.005
	alternative	**-61753.653**	**124057.039**	**0.001**	0.047	Reference	0.000	0.619	21.578	0.749	0.247	0.003
						Test	0.000	0.978	1.156	0.749	0.247	0.003
	PD	-61716.840	123991.464			Reference	0.000	0.604	23.796	0.744	0.253	0.003
						Test	0.028	0.619	18.514	0.701	0.293	0.006
TTG1	null	-39985.635	80514.584				0.000	0.211	2.056	0.728	0.239	0.032
	alternative	-39985.270	80515.880	0.393	0.953	Reference	0.000	0.205	2.136	0.730	0.239	0.032
						Test	0.000	0.221	2.062	0.730	0.239	0.032

PD	-39973.908	80501.255		Reference	0.000	0.458	8.598		0.811	0.179	0.010
				Test	0.000	0.187	1.211		0.732	0.215	0.053
